# A Bio-Inspired, Motion-Based Analysis of Crowd Behavior Attributes Relevance to Motion Transparency, Velocity Gradients, and Motion Patterns

**DOI:** 10.1371/journal.pone.0053456

**Published:** 2012-12-31

**Authors:** Florian Raudies, Heiko Neumann

**Affiliations:** 1 Center for Computational Neuroscience and Neural Technology, Boston University, Boston, Massachusetts, United States of America; 2 Center of Excellence for Learning in Education, Science, and Technology, Boston University, Boston, Massachusetts, United States of America; 3 Institute for Neural Information Processing, University of Ulm, Ulm, Germany; The University of Plymouth, United Kingdom

## Abstract

The analysis of motion crowds is concerned with the detection of potential hazards for individuals of the crowd. Existing methods analyze the statistics of pixel motion to classify non-dangerous or dangerous behavior, to detect outlier motions, or to estimate the mean throughput of people for an image region. We suggest a biologically inspired model for the analysis of motion crowds that extracts motion features indicative for potential dangers in crowd behavior. Our model consists of stages for motion detection, integration, and pattern detection that model functions of the primate primary visual cortex area (V1), the middle temporal area (MT), and the medial superior temporal area (MST), respectively. This model allows for the processing of motion transparency, the appearance of multiple motions in the same visual region, in addition to processing opaque motion. We suggest that motion transparency helps to identify “danger zones” in motion crowds. For instance, motion transparency occurs in small exit passages during evacuation. However, motion transparency occurs also for non-dangerous crowd behavior when people move in opposite directions organized into separate lanes. Our analysis suggests: The combination of motion transparency and a slow motion speed can be used for labeling of candidate regions that contain dangerous behavior. In addition, locally detected decelerations or negative speed gradients of motions are a precursor of danger in crowd behavior as are globally detected motion patterns that show a contraction toward a single point. In sum, motion transparency, image speeds, motion patterns, and speed gradients extracted from visual motion in videos are important features to describe the behavioral state of a motion crowd.

## Introduction

Due to the increasing urbanization, motion crowds become more likely and solutions to prevent accidents or potential hazards as a result of mass panics become more important. Designers of public spaces and intelligent environments begin to consider crowd-dynamics and crowd-environment interactions in everyday situations as well as in exceptional situations such as mass panic [1], [2]. Most public spaces are not equipped with an intelligent crowd management system or automated surveillance system, as suggested in [3], [4].

Research on motion crowds is interdisciplinary. Observing, analyzing, and interpreting data includes the social science, the computer science, and the neuroscience. It involves computer vision to analyze captured video, to characterize, and to formalize patterns of crowd dynamics. Developed metrics (level of service) for crowd dynamics have been described as free, restricted, dense, and jammed people flow [5], [6]. Measures determined during automatic analysis include crowd density, location, and speed; both for individuals and groups of people, as well as estimates of pressure for groups of people. Descriptive models for pedestrian flows are developed on the basis of gas kinetics or fluid dynamics [7], [8]. Research in cognitive science and neuroscience can help to develop assistive tools or an automated analysis of crowd behavior that supports the detection of dangerous behavior. Our goal is to provide a first module of such a system that can process visual motion and extract motion features, including the case of motion transparency as generated by certain crowd dynamics.

Motion transparency is perceived within image regions which contain motion signals in one direction and motion signals in sufficiently distinct direction. As such, motion transparency involves materials that are transparent and the surfaces made of such materials move differently. Likewise, such a percept could be generated through compositions of multiple small opaque parts in the visual field which move differently but coherently in front of a coherently moving background [9], [10]. In our analysis, the notion of motion transparency applies to situations in which the motion signals are generated under limited spatial resolution properties of the sensory acquisition and cortical processing. For instance, motion transparency appears when pedestrians move in different directions within the spatial integration region of a model cell [11].

In our model we use mechanisms that are inspired by the structure and function of the visual system of the mammalian brain. For example, in the primary visual cortex area (V1), simple cells are selective to the light-dark and dark-light polarity of gray-value edges while complex cells are sensitive to oriented edges, independent of their local gray-value polarity [12]. Such contrast edges can be detected by employing local, oriented filters. Examples are Gabor filters [13], which are defined by spatial frequencies, size, orientation, and temporal frequency. They closely resemble properties of contrast sensitive cells in V1. Applying such Gabor filters to videos provides features that are used to establish spatio-temporal correspondences between succeeding frames and, thus, provides initial motion estimates. These initial estimates are further processed in the hierarchy of areas in visual cortex. The middle temporal area (MT) has been described as a major stage for the integration and segregation of visual motion [14]. Our model integrates initial motions in the image plane within a spatial neighborhood for each velocity. Cells in the dorsal part of the medial superior temporal area (MSTd) are selective to large field motion patterns [15], [16], [17]. Our model area MSTd employs different cell types to detect flow patterns of expansion (EXP), contraction (CON), clockwise (CW), and counterclockwise (CCW) rotation as well as linear combinations thereof which result in patterns of spiral motion. Areas MT and MST contain cells that are selective to spatial velocity gradients in visual motion fields [18], [19], [20]. In our model we adopt cells selective to velocity gradients to link their responses to a crowd behavior of slow down which is a precursor for potentially dangerous situations that often develop later in crowds undergoing further periods of congestion and turbulence [2], [6]. Evidences from neurophysiology about the selectivity of cells to optic flow motivated us to develop a model of visual motion processing to achieve a bio-inspired visual, motion-based characterization of danger in motion crowds. Key stages of our model have been developed in the context of figure-ground segregation [21] and the neural representation and processing of motion transparency and binocular transparency [22], [23]. Here, we demonstrate the processing of videos by our model, where these videos show non-dangerous and dangerous crowd behavior.

But how does motion transparency relate to dangerous crowd behavior? Various proposals stress the difficulty of tracking or disentangling individuals in densely packed motion crowds, as they occur in routinely acquired videos, e.g., in subway stations, train stations, in front of counters, at entry and exit locations of public buildings (e.g., [24], [25], [26], [27]). For a low video resolution and a passive camera at high elevation (no zoom, pitch, yaw, nor roll), which is the image acquisition scenario we typically rely on, persons only occupy a few pixels in the image plane. In addition, partial occlusions of persons might occur due to an oblique viewpoint. The small size of a person in the image and the partial occlusion make it difficult to track single persons with their individual movement. Only a large enough spatial resolution allows for the segmentation of items and their motions [6]. Here, we suggest a biologically inspired approach of motion processing that does not require a high spatial resolution. Rather than trying to recover trajectories of single persons, we characterize motions of a larger group of people as they appear as a pattern. Some of these larger patterns contain multiple motions. We define the presence of multiple motions as motion transparency. Such appearance of motion transparency relates in some cases to dangerous crowd behavior.

In our model estimated motions from a group of people are represented by a distribution. This distribution is bimodal in case of motion transparency. To represent bimodal distributions, we use a velocity space that encodes the likelihood of each velocity vector. For instance, a rightward motion of one pixel speed per frame is represented in polar space with the radial component encoding speed and the angular component encoding direction. In this example, the velocity is represented by a Gaussian likelihood centered at zero degree direction and one pixel speed per frame. In this velocity space, we apply a competitive interaction with a positive center Gaussian kernel and negative surround Gaussian kernel to improve the signal-to-noise ratio of encoded motions. Most importantly, this interaction supports multimodal distributions in the velocity space given that multiple motions occur in the video. The spread of the Gaussian in the velocity space is parameterized by the standard deviations along the speed and direction axes, namely *σ_speed_* and *σ_dir_*, respectively. The values for these standard deviations were derived from experimental data, as discussed in detail in [21] and [22]. We visualize the velocity space representation of the motions by reading out the modes of the motion likelihood profile as present for each spatial region encoded by a single MT motion sensitive cell. A flat distribution represents ‘no motion’, a unimodal distribution indicates a ‘single motion’, and a multimodal distribution implies ‘multiple motions’. The presence of multiple motions is equivalent to the presence of motion transparency.

The remaining part of our article is structured as follows: In Section 2 we describe our model and the read-out that we use to visualize the model’s various motion representations. Section 3 visualizes the detected motion of the model when processing generated or recorded videos. Section 4 discusses our model architecture, the detection of non-dangerous and dangerous crowd behavior. We end with conclusions in Section 5.

## Methods

**Figure 1:**
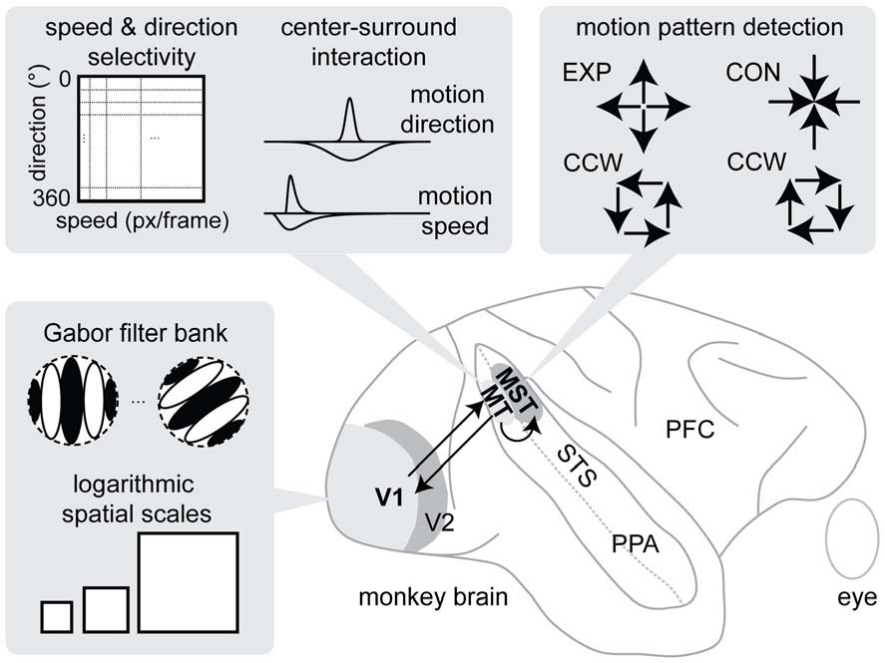
Depicts our biologically inspired proposal of visual motion processing that models the functions of primary visual area (V1), middle temporal area (MT), and medial superior temporal area (MST) as well as their interaction, indicated by an arrow. We consider cells from the dorsal part of area MST, referred to by MSTd, which respond to motion patterns. The three gray boxes show details of these functions. The box to the lower-left shows V1 detecting initial motions from Gabor filtering results at multiple spatial scales. The box to the upper-left shows the motion integration mechanisms of MT that encodes motion in neurons selective to a combination of motion speed and direction (velocity). This velocity space representation is displayed here in a Cartesian coordinate frame for convenience. A center-surround interaction is applied in these codomains of speed and direction. The box to the upper-right shows the globally defined motion patterns that extend over a large region of the visual field and that are detected in model area MSTd receiving input from MT.

Our model architecture, in a nutshell, consists of three model areas located in the dorsal pathway in the primate visual cortex. These model areas correspond to brain areas V1, MT, and MSTd, respectively, see Figure 1. Each model area contains a generic three-stage processing cascade (see e.g., [21], [22], [23]). These stages define an initial filtering, a re-entry stage of modulating feedback (FB), and a stage of competitive interaction. Since in the current model we omitted FB from MSTd to MT and also do not incorporate any FB for MSTd, the description of model areas MSTd and MT simplifies. The next subsection describes model area V1 with its initial motion detection and the three-stage processing cascade. Then, we describe model area MT which operates on a different spatial scale than V1 and also employs a different pattern of local connectivity than V1. Model area MSTd receives input from model area MT and detects motion patterns of large spatial extent. Finally, we describe the read-out of the likelihood representation of motions and their visualization.

### 2.1 Initial Motion Detection in Model Area V1

We describe the varying patterns of structured light in the image sequences using the spatio-temporal function 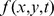 with the spatial coordinates *x* and *y*, and the temporal coordinate *t*. Each image frame of the video is convolved with Gabor filters of different spatial resolution and orientation. Gabor filters resemble the variability of selectivity of area V1 neurons [13]. Movement over time causes a spatial shift of the respective visual structure in a certain direction and for a certain amount. The scalar values of direction and speed uniquely define a spatial shift in the image plane. This vector is typically defined as velocity. Here, the spatial resolution of a Gabor filter constrains the spatial displacement of local, visual structure between frames that can be detected as initial motion velocity estimate. For a proper detection of such displacements we account for the sampling theorem [28]. Thus, for our detector we test spatial displacement of local structure smaller than half of the wavelength of the Gabor filter’s peak frequency selectivity. We use the convolution theorem to improve computational performance. Each image frame 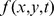 is Fourier transformed with respect to the spatial coordinates. This results in 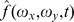, where we denote the Fourier-transformed expressions using a hat-symbol atop. Spatial coordinates *x* and *y* change into angular pixel frequencies *ω_x_* and *ω_y_*. These encode how often a signal change occurs per pixel and the corresponding wavelength specifies the number of pixels that define a full cycle. Note that we do not apply a Fourier transform to the temporal dimension. The spatial Gabor filters in the Fourier domain are defined as
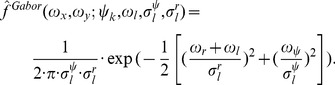
(1)


Equation (1) defines a parameterized Gaussian kernel of unit integral that is centered at the frequency *ω_r_+ω_l_* in radial direction and *ω_ψ_* in angular direction with their respective standard deviations *σ_l_^ψ^* = 1.5•*ω_l_•sin(Δψ)* and *σ_l_^r^* = 1.26•*ω_l_•(β-1)/(β+1)*. The factors 1.5 and 1.26 define the overlap between neighboring filters and have been chosen to improve the uniform coverage of the frequency domain by these Gabor filters. The angular difference *Δψ* = *180°/N_ori_* is defined using the number of orientations *N_ori_*. The orientation of Gabor filters is defined by the angle *ψ_k_*. In order to account for this orientation of Gabor filters, we apply the following rotation transform 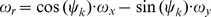 and 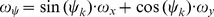
to the Cartesian system used for the Fourier transform.

As pointed out above the modulation that the spatial frequency selectivity of a Gabor filter imprints a modulation to the image signal. This constrains the maximal displacement (or speed) of local image structure that can be reliably detected. In order to detect motions with different speeds (and direction) in the image, we apply a filter bank of spatial frequency selectivity and spatial orientations. In our definition, we conveniently express the motion speeds 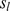 in the image as multiples of the minimally detectable speed 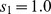
pixel/frame which references the smallest Gabor filter. The speed scaling scheme is defined by 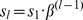 (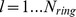) in which individual speeds are linked to wavelength 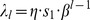 (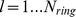, 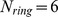, 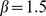). For this relationship, we assume that the displacement *Δs* between frames is less or equal to half of the wavelength *λ*, which the Gabor filter is maximally tuned for. Thus, the factor *η* ≥2 frame, here set to *η* = 2.5 frame. For motion directions, we correlate image phase for all orientations 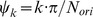 (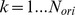, 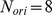). These orientations are indexed by *k*. The angular frequency 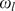
for the Gabor filters is defined by 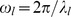
• 1pixel/frame. Note that a normal flow detector would test only the motion orthogonal to an extracted orientation.

The filtering of an input signal with the above defined Gabor filter kernel from Equation (1) is computed by using a multiplication in the Fourier domain. The symbol *F^−1^* denotes the inverse Fourier transform. Its application yields the complex numbers:
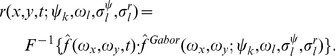
(2)


These complex numbers are used to define an amplitude and angle. Our motion detector uses only the angle also referred to as the local image phase 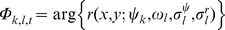. We only use the image phase signal because it is largely insensitive to the overall brightness changes in the spatio-temporal image sequence. Note that evidence suggests that some V1 neurons encode local image phase [29]. Initial motion is calculated with the extended Hassenstein-Reichardt detector [30] using local image phase values as input. Therefore, a local image phase
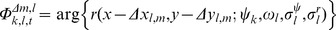
(3)is spatially shifted by the horizontal 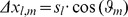
and vertical 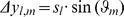 offset and is compared against the non-shifted, but temporally delayed phase. The forward correlation from time *t_0_* to *t_1_* is defined by



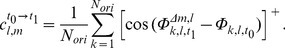
(4)The rectified cosine-function defines the tuning between phase differences and 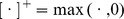 denotes a half-wave rectification to avoid negative amplitudes in the responses. After rectification the correlation results from all orientations are summed. The backward correlation 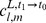 is analogously defined by inverting the temporal order, correlating signals from frame captured at *t_1_* with those from the frame captured at *t_0_*. We do not want to detect flicker motion that is characterized by the simultaneous appearance of forward and backward correlations. Thus, we subtract forward and backward correlations.
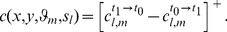
(5)


This subtraction results in a positive response in cases of coherent motion along a given direction. Next, we describe the mechanisms of the model area V1 that take the above correlation result from Equation (5) as input.

### 2.2 Three-stage Processing Cascade for Model Area V1

The three-stage processing cascade is motivated by the layered cortical processing of the visual cortex. This cascade utilizes model cells or groups of cells as the basic computational unit. Their response behavior can be characterized in accordance to their biophysical properties in terms of changes in membrane potential (voltage) when a cell receives excitatory or inhibitory input from other cells [31]. For our computational analysis, we consider the membrane potentials of a group of cells as mean activation level. Since we abstract from biophysical and neural details of the model components, we use the term activity or activation throughout the rest of the paper in order to denote the responses of stages in our model.

The *first stage* includes a spatial integration and nonlinear signal enhancement defined by the ordinary differential equation (ODE):
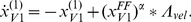
(6a)


The signals 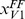 and 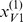 denote model activities of the input signal which is the output of the initial motion detector, see equation (5). These initial motions are considered as driving feed forward signal. Formally, we have 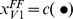 and 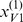 is the activity of the integration stage. In this stage, a quadratic function, *α* = 2 in equation (6a), is applied to model a nonlinear signal transfer applied to the motion detection outputs, which compresses very low responses and amplifies higher level activations. After applying the nonlinear transform, the signal is convolved with the Gaussian filter *Λ_vel_*, the convolution operating on motion speeds and directions. We interpret this convolution as interaction between model cells encoding similar motions. In our implementation we solve all ODEs, such as defined by equation (6a), by using their steady-state solution. Iterations between different frames of the input sequence are iterations of these steady state solutions assumed for each individual frame.

In the *second stage* of the cascade, FB modulates driving forward signals. Feedback originates from a visual area that is located higher in the processing hierarchy than the one that is generating the feeding signal. Activities in model area V1 are modulated by activations accumulated in areas higher up in the hierarchy concerned with motion (e.g. MT) and form processing (e.g. V2, V4). This FB is represented at a lower spatial resolution than the signal of the area which it is feeding into. Thus, FB helps to disambiguate local motion which can be ambiguous due to the aperture problem. It also enables signal propagation across modeled visual space. We combine driving and FB signals through modulation: Signals that encode the same motion are enhanced while others remain the same. To be effective, a FB signal always requires a driving forward signal; however, FB alone cannot generate any signal enhancement. In formal terms, the signal 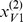 is formed by a non-linear combination of the FB signal 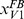 and the driving forward signal 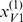 from the first stage
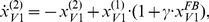
(6b)where *γ* is a parameter that typically ranges between 10 and 100 to amplify FB. Typically, values of 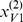 range between zero and one. All equations and parameters appear without units, as we did not aim for modeling the exact biophysical process of axons, synapses, or neurons.

In the *third stage* of the cascade, signals are normalized by dividing activity of a target cell by the activation summed over encoded motions in a velocity neighborhood of the target cell. This division keeps signal amplitudes bounded. In combination with the FB modulation of the second stage, this normalization realizes a biased competition that deemphasizes signals which encode features that did not receive any enhancement by FB. Formally, the signal 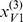 integrates the signal 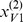 of the second stage by summing over all encoded motions, respectively, for each spatial location which yields:
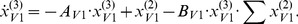
(6c)


The symbols *A_V1_* and *B_V1_* denote constant parameters which control the strength of normalization. Typically, parameter *A_V1_* has a value of 0.1 and values of 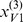 range between zero and one. Values for parameter *B_V1_* are approximately one. The steady-state solution of this Equation (6c) is 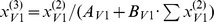, which shows the normalization property. Due to the division by the sum, assuming *B_V1_≥1*, values of 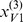 will not exceed the upper limit of one. Since the sum ranges over all velocities, this normalization favors a single motion vector. This is different for model area MT, which we describe next.

### 2.3 Three-stage Processing Cascade for Area MT

The *first stage* uses an isotropic integration of signals from model area V1 to model area MT. During integration the spatial resolution is reduced by a factor of five which we link to a change in receptive field (RF) size between V1 and MT cells. The RF denotes the region in visual space (or in the image domain) in which an input stimulus leads to a response of the target cell at a fixed spatial reference position. In cortex the visual input is mapped to representations in several areas in a visuotopic fashion, i.e. preserving the neighborhood relations. For example, such a mapping from the retina to the primary visual cortical area V1 resembles the high spatial resolution at the center of view (leading to a magnification of the fovea) and lower resolution in the periphery. In the application here, the relevance lies in the fact that these two factors are different and must be considered in conjunction in order to derive proper filter sizes to be used in model implementations. The RF size in MT is about ten times larger than that in V1 [32]; however, the magnification factor in V1 is about one-fifth of that of MT [33]. This difference between magnification and RF size might account for the Nyquist sampling theorem, where MT applies a low-pass filter which restricts the highest spatial frequency to at least half of the sampling frequency [28]. In our implementation the down-scaling of spatial resolution in representations is achieved in two steps. First, the input velocity space is convolved with a Gaussian filter of appropriate size to meet the sampling theorem [28]. We illustrate “appropriate” with the following example: Assume that the maximum normalized frequency is π. Then the power in the spectrum of the Gaussian filtered signal with σ at the sampling rate *r* is *σ*/√(2*π*)•exp(-(*π*/*r•σ*)^2^/2). For instance, for the sampling rate *r* = 5 we apply a Gaussian filter with *σ* = 5 pixel the power is exp(-*π* ^2^/2) 100% = 0.72% of the maximum power *σ*/√(2*π*). For the sampling of V1 for MT activity, we use the same numbers as in the example. Accordingly, and second, samples for every fifth value of the filtered motion signal are selected. In cortex, integration in MT leads to an increase in the direction tuning bandwidth (mean value of 95°) compared to that of V1 (mean value of 68°) [34]. This increase of bandwidth is an indicator for the integrative or smoothing behavior of cells in area MT. In formal terms this integration is denoted by.
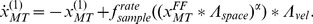
(7a)


Motion signals from model area V1 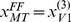 are spatially integrated by convolution with the Gaussian 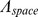 and subsequently sampled using 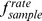, a linear interpolation for arbitrary sampling rates. Then, the signal is exponentiated by *α*, like in the Equation 6a. Finally, the sampled signal is smoothed in the velocity domain by convolution with the Gaussian 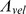.

In the *second stage* of the cascade, FB from other model areas, such as MSTd ( [22], see below) could be included, in principle. Since we did not further investigate the tuning of cell responsiveness in MT driven by FB, the second stage response is denoted by the identity 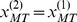 in its steady state.

The *third stage* of the cascade, calculates local center-surround interaction between encoded motions. This interaction supports similar motions and suppresses dissimilar ones. Formally, the signal 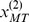 is convolved with 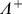, a Gaussian filter that is defines the support from similar motions in the velocity space. The inhibitory field is computed by convolving the signal 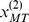 with the Gaussian 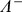 that defines a field of local suppression. Typically, this filter kernel connects to dissimilar motions. The supportive and suppressive fields are combined in a competitive scheme that uses divisive inhibition:
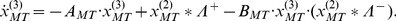
(7c)


The value of parameter *A_MT_* is typically set to 0.1, where values of 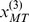 range between zero and one. Values for parameter *B_MT_* range between 1 and 10. As defined in Equation (7c) this ‘soft’ competition between neighboring velocities enables the representation of multiple velocities. Unlike the normalization mechanism in model area V1 that favors the encoding of a single motion. This ‘soft’ competition in model area MT is the main difference to previous models of motion processing [35], [9], [36], [10], [37], which leads to the desired properties that motion likelihood values in the velocity representation can coexist, given that speeds and directions are “sufficiently different”. This is the prerequisite of representing motion transparency as generated by specific motion crowds.

### 2.4 Detection and Processing of Motion Patterns in Area MSTd

The dorsal part of medial superior temporal area (MSTd) contains cells selective for large field motion patterns [15], [16], [20]. Such patterns are, for instance, generated on the retina during self-motion. Movement along the direction of gaze results in an expansion motion field with radially outward pointing vectors. We call such a pattern a source, since people move in all directions departing from the same spot. Movement in the opposite direction of gaze gives a contraction motion field with vectors radially inward pointing. This, we call a sink. Sinks observed in motions of crowds relate to potential danger as they describe a compression. A source or sink in the image plane can be at various locations (*u, v*). Points in the image plane are referenced to by (*x, y*). We define the angle *ψ*(*x-u, y-v*) = arctan2(*y-v*, *x-u*) with respect to these source or sink locations (*u, v*). This angle is used to define pattern flow vectors (cos(*ψ*), sin(*ψ*)) at locations (*x, y*) in the image plane. For rotations of this local angle *ψ* by an additional amount *δ*, we define motion patterns of EXP, CON, CW, CCW, and combinations thereof. For instance, *δ* = *π*/2 defines a CCW motion pattern. Such motion patterns are encoded in likelihood values for motion directions *φ* defined by.
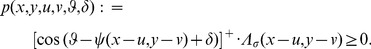
(8)


We use the half-wave rectified cosine-tuning [cos(•)]^+^ to define likelihood values for motion patterns. Multiplication with the Gaussian *Λ_σ_* weighs the influence of a motion pattern less the further distant this motion is from the location (*u, v*). The parameter *σ* denotes the standard deviation of the Gaussian and is set to 80% of the visual field. We use these pre-defined motion patterns from Equation (8) within the previously described three-stage processing cascade.

The *first stage* of the cascade, the forward signal integration, is described by:
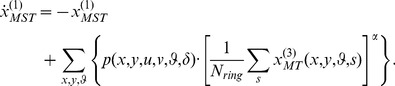
(9a)


The mean value of the signal 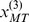 for all speeds is computed, non-linearly enhanced (*α* = 2), and then projected onto the pre-defined motion patterns. This projection is computed by the inner product that ranges over the dimensions *x*, *y*, and *φ* and defines likelihood values for motion patterns. The first-level Equation (9a) temporally integrates these pattern likelihoods using the variable 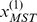. This variable depends on all sampled image locations (*u*, *v*) combined with all sampled patterns *δ*.

The *second stage* of the cascade again allows for an integration of an FB signal which modulates the pattern likelihoods. Since we did not incorporate a higher-order bias to enhance the responses of motion pattern cells, the FB signal is set to zero and we use the identity 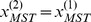.

The *third stage* of the cascade in model area MSTd normalizes likelihood values with respect to all spatial locations (*u, v*). To simplify, the center kernel takes directly the feeding signal 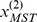, and the surround computes the mean over all spatial locations. Center and surround signals are embedded into a competitive scheme, expressed by:
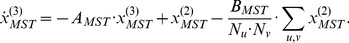
(9c)


The 2^nd^ term in Equation (9c) defines the center or supportive signal and the 3^rd^ term the surround or suppressive signal. In this case, we use the surround to normalize likelihoods. The remaining identifiers *A_MST_*, *B_MST_*, *N_u_*, and *N_v_* are parameter values. Table 1 lists all model parameters and their values that are kept constant during simulations.

**Table 1:** Parameter values for initial motion detection and three-stage-processing cascades for signal integration in model areas V1, MT, and MSTd.

Description	Value	Eq.
Detection of initial motion		
Orientations	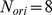 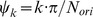 , 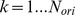 ,	1
Rings of the Gabor bank	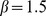 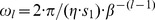 , 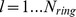 , 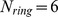 ,	1
Overlap factors	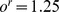 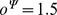 and	1
Radial standard deviation	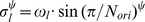	1
Tangent standard deviation	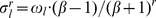	1
Speed to wavelength factor	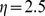 ( 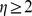 due to the sampling theorem)	1
Motion directions	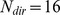 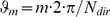 , 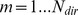 ,	3
Motion speeds	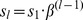 , 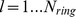 , 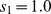 pixel/frame	3
Three-stage processing cascade of **model area V1**		
Nonlinearity α	2	6a
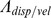 Gaussian filter	Motion speed: 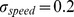 pixel/frame and 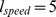 pixel/frame a Motion direction: 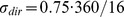 ° and 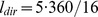 °^a^	6a
Boundary conditions	Motion speed: Neumann. Motion direction: Circular	6a
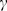 Feedback constant	100	6b
Normalization A_V1_	0.01	6c
Normalization B_V1_	100/112	6c
Three-stage processing cascade of **model are MT**		
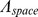 Gaussian filter	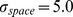 pixels and 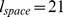 pixels^a^	7a
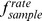 Sampling rate	5	7a
Nonlinearity α	2	7a
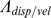 Gaussian filter	Same as in V1	7a
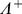 Gaussian filter	Motion speed: Dirac pulse. No kernel is applied. Motion direction: 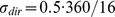 ° and 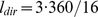 ° a	7c
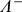 Gaussian filter	Motion speed: 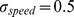 pixels/frame and 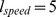 pixels/frame a Motion direction: 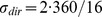 ° and 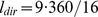 ° a	7c
Boundary conditions	Motion speed: Neumann; Motion direction: Circular	7c
Normalization A_MT_	0.01	7c
Normalization B_MT_	10	7c
Three-stage processing cascade of **model area MSTd**		
Positions of pattern (u, v)	□ {(0%, 0%), (25%, 0%), (50%, 0%), (75%, 0%), (100%, 0%), (0%, 50%), (25%, 50%), (50%, 50%), (75%, 50%), (100%, 50%), (0%, 100%), (25%, 100%), (50%, 100%), (75%, 100%), (100%,100%)}	9a
Parameter for pattern δ	□ {0°, 45°, 90°, 135°, 180°, 225°, 270°, 315°}	9a
Nonlinearity α	2	9a
Normalization A_MST_	0.01	9c
Normalization B_MST_	10	9c
Pattern numbers N_u_, N_v_	N_u_ = 5, N_v_ = 3	9c

a The length specifies the size of the support for the filters.

### 2.5 Read-out and Visualization of Encoded Velocities

All model areas use an explicit likelihood encoding for motion. Our model includes three hierarchically organized areas for the detection and integration of motion likelihoods, namely V1, MT, and MSTd. In the first two model areas the likelihoods of local velocities are encoded for different spatial detail and resolution. The area MSTd encodes likelihoods for specific motion patterns, or configurations of spatial flow. Velocity likelihood values are read out from their respective representations for display purposes by applying an iterative method. Velocity representations are defined for each spatial location and encode the direction and speed of a particular motion in the image. In the case of opaque surfaces or crowds that move coherently in one direction, the velocity space should contain a single peak of activation. This peak defines the maximum likelihood estimate for the current motion estimation. When multiple motions occur, as in the case of transparent motion, the velocity space representation contains multiple peaks. These are read out in an iterative fashion. The first step of the method locates the maximum amplitude of the responses in velocity space. If the maximum is below a certain threshold value, ‘no motion’ is returned. Otherwise, a discretized Gaussian is positioned with its mode at the location where the maximum appears in the velocity space. The inner product between the Gaussian weights and the filter responses for the corresponding velocities is computed. The resulting sum of weighted motion responses is normalized by the sum over the products of Gaussian entries and signal values. In all, the read-out procedure computes a weighted vector average of the velocities, given the filter responses and the Gaussian weighting coefficients. If multiple motions are present in an image patch, this procedure is iterated so as to continue the selection and Gaussian weighting on the next peak in the velocity space. In particular, the next step suppresses all likelihoods that were read out during the previous iteration step and set them all to zero. Then the iterative method continues with the first step by anchoring the Gaussian weighting function at the next peak. Again, the average motion response is determined by the projection of filter amplitudes of velocity selectivity to the Gaussian weighting function. This is iterated until we reach a specified number of motions. Here, we consider two motions that may exist in an image patch displaying a group of people. For the read-out procedure we use the encoded velocity from the last stage in model area MT, namely 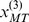.

The next computational procedure converts the above acquired vector fields into a response field map which contains ‘no motion’, ‘single motion’, or ‘multiple motions’ entries in the spatial resolution of model area MT. Regions with no motion are encoded as black, regions with a single motion are encoded as gray, and those with multiple motions are encoded as white. These maps are interpolated to match the spatial resolution of the original video frames using a nearest neighbor interpolation. This interpolation maintains the original encoding into ‘no motion’, ‘single motion’, and ‘multiple motions’. We temporally filter these higher resolution maps that the encoding remains only if it is constant throughout three successive frames. Otherwise, the corresponding pixel location is assigned ‘no motion’.

Motion pattern likelihoods from model area MSTd are not read out. For their display, we simply normalize with respect to their maximum value within a population to report a normalized activation signal.

## Results

Our model was originally designed to explain animal and human data to closely model cell responses and behavior when processing motion transparency [22], [38], [11], [39]. Since the model successfully explains a wealth of perceptual data, we decided to apply the framework directly to videos of crowds. It is expected that the model mimics the response characteristics of a viewer. In the following subsections, we describe the model’s simulation results when processing synthetically generated or recorded videos. We selected data sets to probe the model’s capability in processing videos that show crowds of moving people from varying distances. Here, we used simulated crowd data for a virtual camera setup that models the situation of a realistic camera setup monitoring spaces where motion crowding might occur. Therefore, we do not have access to calibration data that defines the exact viewing geometry in terms of the distance, viewing angle, and spatial image resolution. We only have a rough estimate of distance measure from the pixel resolution and an average height of people in the image. In a first set of videos individuals are a few pixels high in the image. In another set of videos persons are 80–40 pixels high. We also used point sets (random dot kinematograms) that move in space-time viewed from above to study the clustering and collective movement patterns when crowds are forced to pass through a narrow passage. These sequences were generated based on the social force model of crowd dynamics.

### 3.1 Non-dangerous Crowd Behavior Shows Motion Transparency

In this scenario, we synthetically generated videos using the social force model [7], [40] to simulate crowds seen from above. In a nutshell, this model expresses the interaction between individuals within a group of pedestrians as well as individual’s interaction with boundaries, e.g. walls. This interaction employs “social” and physical forces in a many-particle system. Walls and other pedestrians act as repellors. Each pedestrian has a goal direction that defines an attractive force, which it follows with a maximum walking speed. Taken together, the linear superposition of forces describes the velocity and position change of each pedestrian in the model.

**Figure 2:**
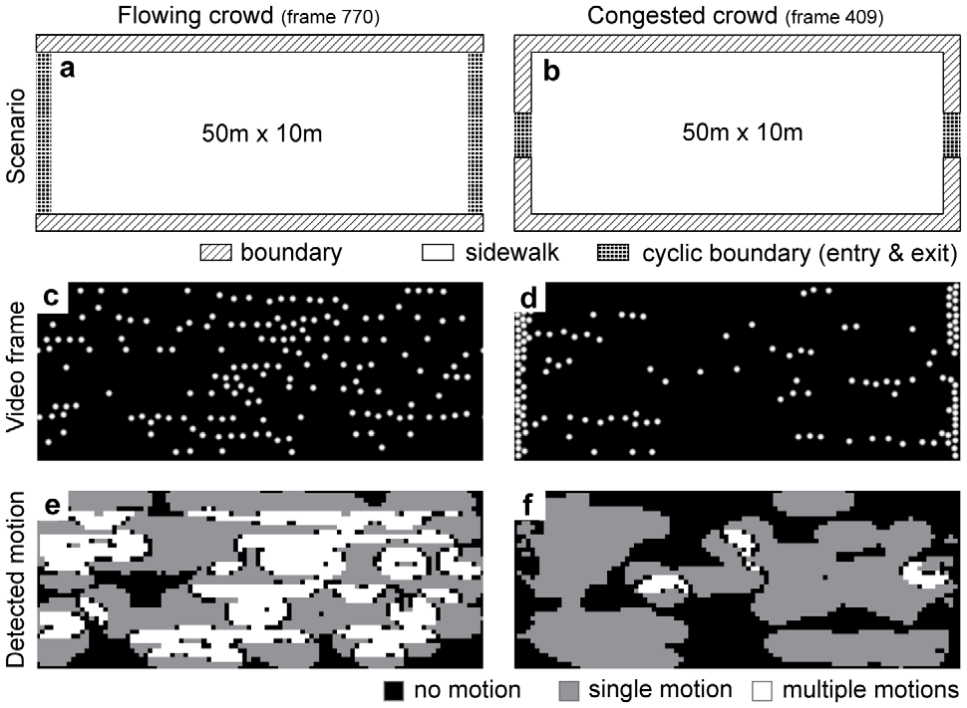
Shows motion transparency for the scenario of flowing a) and not for the scenario of congested crowd motion b). These two scenarios were simulated using the social force model [7] where pedestrians are modeled as dots and viewed from above (birds-eye view). Panels **c)** and **d)** show image frames of the generated videos for a flowing and congested crowd, respectively. The detected motion for these frames is shown in **e)** and **f)**. A legend at the bottom of the figure denotes the gray-value encoding of motion.

In the first setting of Figure 2a and 2b approximately 200 pedestrians are simulated to walk on a sidewalk that is 50 m long and 10 m wide. We employ a cyclic boundary condition in horizontal direction. Half of all pedestrians go to the left. The other half goes to the right. The parameters of the social force model are the same as suggested by Helbing et al. [7]. Note that the social force model that describes crowd dynamics was introduced in [40] and, later, was extended in [7] to describe panic in crowd escapes. Due to its evolved description, we used the formalism of [7] to generate reference scenarios of crowd dynamics and to probe our model. The simulation with the social force model for this first setting shows a loose organization of pedestrians into eight lanes of alternating motion direction (Figure 2c). Processing this generated video, our model detects large regions of motion transparency, encoded in white in Figure 2e. In an altered setting, we closed the ends of the walkway by introducing additional walls leaving only one meter wide exits and entries (Figure 2b) while maintaining the cyclic boundary condition for these exits and entries. In this setting, pedestrians clog along the vertical walls, see Figure 2d. Processing the generated video by the model shows only a few small regions of motion transparency (Figure 2f). These regions disappear at future steps as will the regions with a single motion. The two crowds stop moving coherently. This is already indicated in Figure 2f where regions close to the vertical borders appear motionless. The repelling forces between individuals of the crowd increase steadily and can potentially lead to injuries of individuals. These two settings exemplify: Non-dangerous crowd dynamics with opposite motions appears as motion transparency in videos and dangerous crowd dynamics is depicted in a transition from motion to no motion or the stop of movement in the event of clogging.

**Figure 3:**
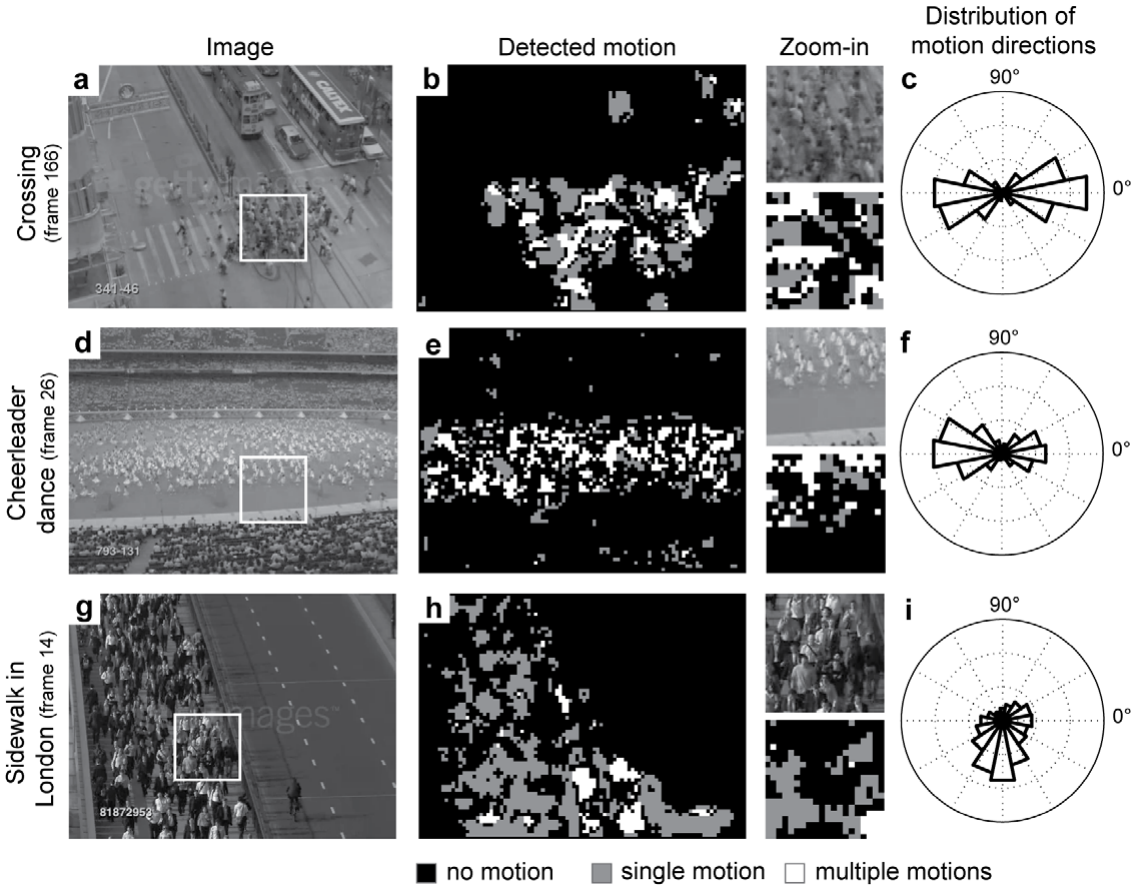
Provides examples of motion transparency detected in image regions of videos showing a crossing (1^st^ row), cheerleader dance (2^nd^ row), or sidewalk in London (3^rd^ row). The first two examples a) and d) have a bimodal distribution of motion directions which indicates motion transparency for the zoomed-in areas in image frames of b) and e). Panels c) and f) show a pixel-wise labeling of ‘no-motion’, ‘single-motion’, and ‘multiple-motions’ for these two examples. The third example, the sidewalk in London, shows a single motion indicated by the unimodal distribution of motion directions in g) pooling over the zoom-in region of the image shown in h). The pixel-wise assignment gives i).

We applied our model to real-life videos showing non-dangerous crowd behavior to verify the occurrence of motion transparency. The first video shows pedestrians on a crosswalk walking along opposite directions while organizing into lanes, Figure 3a and 3b. This example shows that multiple motions are present in various regions, in particular in the zoomed-in regions. This is also visible in the distribution for motion directions pooled from the zoomed region that is shown in Figure 3c. The second video shows a cheerleader dance, Figure 3d. The zoom-in region shows that motion transparency occurs within the region of the field where the cheerleaders perform, also visible in the distribution of motion directions in Figure 3f. No motion appears in the empty region in the lower half of the zoomed-in patch. Motions are detected in the audience because they do not appear purely stationary, instead they move, wave, and cheer. The third video shows a busy sidewalk in London, Figure 3g. Only a few pedestrians walk toward the camera. These appear mainly to the right-hand side of the sidewalk from the camera’s perspective. Because of the main upward-stream of motion only a marginal area within the zoom-in region appears as motion transparency, Figure 3i. The distribution for motion directions is unimodal. The first two examples of the crosswalk and cheerleader dance illustrate that motion transparency appears in real settings and can be reliably detected by our model. The example of the London sidewalk shows that non-dangerous crowd behavior does not always produce motion transparency. This observation indicates that the feature of motion transparency alone is not a stable indicator for danger in crowd behavior. In the next examples we will study the motion generated at crosswalks with a different density of people.

**Figure 4:**
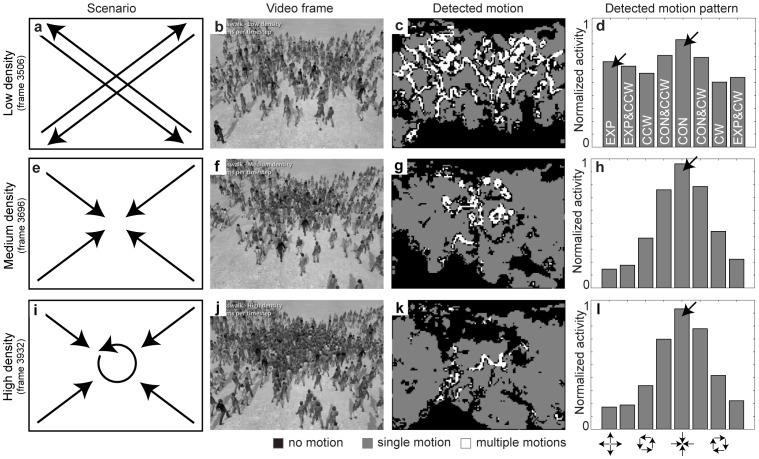
Shows the detected motion for a crosswalk of varying people density [41]. **a)** Shows the scenario and motions for a low density. **b)** A single image frame from the video. **c)** The detected motion shows large regions with motion transparency. **d)** Globally detected motion patterns indicate a motion pattern of EXP and CON. **e)** As the density increases people radially stream inward indicated by the arrows. **f)** An image frame for increased density. **g)** The regions that contain transparency are reduced. **h)** A strong motion pattern of CON is present. **i)** For a further increase of density the motion pattern changes: The central part shows a spiral motion, that of joined and exiting motions from four sides – like in a rotary. **j)** An image of the video for an even further increase in density. **k)** Fewer parts exhibit motion transparency. **l)** Patterns of CON and CON&CW are active. The latter pattern captures some of the rotational inward, spiral motion in the central part of the image. All motion patterns refer to the center of the image plane.

For the simulation of different people densities at a crosswalk, we use rendered videos from Narain et al. [41]. These videos show a simulation of crowd behavior as a whole modeled by “unilaterally incompressible” fluid flow evaluated on a grid. It should be noted that various physics-based models of fluid dynamics or particle systems have been deployed to model crowd dynamics. In our model simulations we use video material that was generated with the social force model or fluid-dynamic model, see Narain et al. [41]. Although these two models vary in detail, they both simulate mass behavior which we use to detect critical visual features. Simulated individuals in this scenario have a height of 80–40 pixels for image frame resolutions of 640×480 pixels. The first scenario in Figure 4a shows two pairwise intersecting people streams for low densities as indicated by the arrows. Figure 4b shows an image frame of the processed video. For this low density people can cross as indicated in Figure 4a which leads to motion transparency encoded as white regions in Figure 4c. This is also indicated by the simultaneously detected CON (contraction) and EXP (expansion) motion patterns in Figure 4d. A sink and source point are detected with the sink point being stronger which suggests an accumulation of people in the center. The simultaneous presence of these two motion patterns suggests that the two people streams are indeed intersecting as schematically drawn in Figure 4a. Increasing the density of people leads to a clogging at the intersection of the people streams that come from four different directions, see Figure 4e (compare with the discussions in [2], [6]). In the depiction of the scenario all arrows point inward. Motion transparency remains to exist close to the center patch (see the white encoded areas in Figure 4g). However, the people in the center, although clogged, still keep moving. This corresponds to the observed turbulences in the flow pattern [2]. In this case the model MSTd detects a strong CON motion pattern, see Figure 4h. This means a sink point in the people flow is detected and people accumulate in the center. For an even higher density the clogging intensifies and the motion speed in the image plane is reduced. Figure 4i shows the scenario where the center part of the crowd exhibits a rotation and people from all four directions join this center part. The corresponding detected motion categorization is shown in Figure 4k. Only at the border where people transition from a radially inward motion into a circular motion, motion transparency is detected. Not only the CON motion pattern is detected but also the combination of a CON & CW (clockwise rotation) motion pattern responds stronger than in the medium density scenario, compare Figure 4h (medium density) with Figure 4l (high density). This seems counterintuitive compared to the counterclockwise rotation present in the video and indicated in Figure 4i. But due to the perspective view during the image acquisition the image region that contains the rotation pattern appears as an elliptic pattern. In addition, the center of rotation is shifted upward to the top edge of the screen. Figure 4i is only a depiction and does not reflect these details. However, the cell responses are reported with respect to the center in the image plane. Thus, a small response for clockwise rotation originates from the people stream coming from the lower, left image corner that is combined with the people stream from lower part of the rotatory, which moves to the right. These two motions are compatible with segments of a CW motion pattern roughly aligned with the center of the image plane and, thus, in the model the CON & CW motion pattern responds slightly stronger compared to the CON & CCW (counterclockwise rotation) motion pattern. To sum up, we investigated different appearances of spatio-temporal crowd configurations and the motion features that characterize them. We particularly investigated the role of motion transparency as characteristic feature of crowd behavior in videos. Motion transparency occurs for a low density moving crowd that is crossing. This is identified by the appearance of multiple motions in a small region of image space that is covered by the size of spatial motion filters in the model. We emphasize that the detection of multiple motions necessitates a model mechanism that allows for a simultaneous representation of several motion directions and/or speeds at a single location. This is not achieved by classes of motion models in which a unique interpretation of local measures is enforced so that the mechanism has to 'decide' for the most dominant velocity that might be a mixture of several underlying motions. The appearance of motion transparency continuously decreased with an increased density of people in which the appearance of dominant motion components in a spatial neighborhood cannot be detected robustly. The spatial organization of these flow patterns gives rise to particular motion patterns, such as CON and CW or CCW motion patterns and their superposition. The contraction of the spatio-temporal patterns is indicative of an increase in crowd density which is accompanied by swirling or rotation motions in either direction. These observations support our claim that patterns of motion appearance manifest in motion transparency and in particular in the combined presence of spatio-temporal motion patterns. The analysis of such features allows for an identification of crowded motion where multiple speeds or directions occur, which eventually leads to critical situations in the overall behavior of the motion crowd.

### 3.2 Motion Transparency Occurs at Junctions in Hallways with Moving People

**Figure 5:**
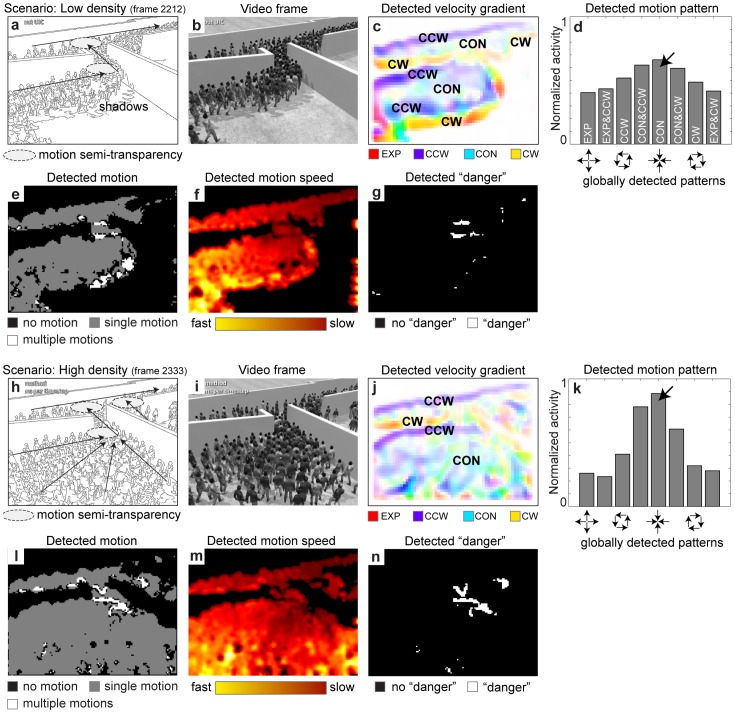
Shows the analysis of a simulated evacuation scenario. **a)** For low density two 90° turns in the main motion direction appear. **b)** An image frame of the video displaying the evacuation. **c)** Velocity gradients represent local motion patterns of EXP, CCW, CON, CW, and combinations thereof. Boundaries between motion and no motion appear mainly as local CCW and CW and CON is detected locally for the people streams which indicates their slow down or negative speed gradient (deceleration). **d)** Globally detected motion patterns show a weak activation of the CON pattern that is centered in the image plane. **e)** Motion transparency appears at the narrow passages and 90° turns. **f)** At the same regions the image speed is slow. **g)** A combination of transparency and slow speeds shows “danger zones”. **h)** At high density the lower room is completely filled with people. **i)** An image frame of the video with higher people density. **j)** Local motion patterns of CW and CCW appear at boundaries of motions and the pattern of CON is now spread over the entire area that corresponds to the lower room. **k)** This increased area that has an CON velocity gradient yields an increased activation of the global CON motion pattern compared to the activation of the CON pattern for low density in d). **l)** Again, motion transparency appears at the narrow passages. **m)** Motion speeds are slow at the passages. **n)** “Danger zones” for the high density scenario.

Another critical scenario appears in hallways during an evacuation [2]. For the model analysis we again use rendered videos from Narain et al. [41] that show the evacuation of a building, especially the people flow at a junction in the hallway (compare also with the simplified scenario in [7]). Figure 5a shows the scenario for a low density of people and Figure 5b a single frame of the video. Due to the merging people stream and the turns, there is a slow-down before the narrow passage that leads to the exit. This slow-down is characterized by a negative speed gradient. We recently proposed a model for the detection of velocity gradients which includes speed gradients [42]. This model uses the MT motion representation to compute directed, spatial velocity derivatives that are registered within a local gauge coordinate system, which in turn is oriented along the local flow direction (for tangential flow derivatives) or perpendicular to it (for the flow derivatives in normal direction). The computation of velocity gradients is based on spatial derivatives of the vector flow field. These derivatives are computed based on the representation of flow vectors by likelihood values in the velocity space. The derivative tangent to the local flow direction is computed by taking the differences of activities encoded in velocity spaces at spatially offset positions along the axis orthogonal to the direction of the encoded velocity. The derivatives along the normal direction are computed analogously. For instance, the expansion flow that is generated when walking toward a fronto-parallel plane has a positive speed gradient along the local flow direction. We compute the flow derivatives for the detected motion and show them in Figure 5c. Their direction is encoded in colors ranging from red (EXP), to violet (CCW), to light blue (CON), to yellow (CW), and again to red. We associate these locally detected velocity gradients with global pattern motions because these global motion patterns locally show these gradients. To help the reader, we labeled regions of velocity gradients according to their color in Figure 5c. The upper hallway has a constant slow-down in motion which is indicated by CON. This CON is in part due to the camera’s perspective. Another region of slow-down or CON exists before the turn. Other velocity gradients (CW and CCW) mark boundaries between regions that contain motion or not. Not only is CON detected locally by a decrease in motion speed, but it is also detected over a large region of the field of view. Figure 4d shows an CON motion pattern centered in the image. Thus, CON is detected both locally and globally. Velocity gradients in prior scenarios have a similar characteristic in the 1^st^ scenario, or are absent in the 2^nd^ scenario, see Figure S1, or are difficult to interpret in the 3^rd^ scenario, see Figure S2. Additional information is provided in the supplement Text S1.

We further evaluated features of motion transparency and image speed. In this evacuation scenario the people stream makes several abrupt turns of 90° in order to leave the space. Our model detects motion transparency at three locations in the image, see Figure 5e. Two of these three locations correspond to the entry and exit point of the narrow passage from the room into the hallway. These are the locations where the stream of people makes the previously described 90° turns and, thus, need to slow down, get slightly compressed, and finally joins a second stream of people. Due to the spatial integration mechanisms the model represents the turning and merging streams as regions that contain motion transparency. Motion transparency is also detected at the location of the shadows on the ground. Arm movements appear during half of the walking cycle to move backward while the main body trunk is moving forward. In the other half of the cycle arms go forward but faster than the body trunk. A 180° directional difference in motion appears in combination with a motion speed difference. Our model integrates these motions and represents them at one spatial location as motion transparency due to their proximity in the stimulus (lower right part in Figure 5e). Figure 5f shows the motion speeds encoded in colors from yellow to red to encode fast and slow speeds, respectively. We chose the red color to encode slow speed to indicate danger. Dark red regions appear at the narrow passage. Motions on the right image border are artifacts due to assuming circular boundary conditions for the detection of initial motion signals.

Regions in crowds which may give rise to potential hazards are indicated by the combination of features. If slowing-down motion speeds occur in combination with motion transparency, this indicates a decreased throughput and, thus, increased pressure. A “danger zone” is detected. We employ a simple decision-rule for the detection of such “danger” zones combining transparency with slow speeds as is shown in Figure 5g.

The scenario for a high density of people shows Figure 5h and 5i. For a high density the empty space in the lower-left room is completely filled with people. Detected velocity gradients indicate a large region of CON, essentially in the entire space of the lower-left room, see Figure 5j. Due to the larger area of CON compared to Figure 5c, the activation of the CON pattern in the center of the image which Figure 5k shows is stronger than that in Figure 5d. This indicates a slow-down of the people stream in front of the narrow passage. Detected CON occurs in the spatial neighborhood of regions in the image plane that contain danger for the crowd, here, the narrow passage. Main regions of motion transparency occur at the different entry and exit points of hallways according to Figure 5l. In addition regions of motion transparency appear in the upper hallway on the left end. This is due to the arm movements that have been described previously. Figure 5m shows the detected motion speeds. Again speeds appear slowest next to the narrow passages and within these passages. The combined occurrence of slow motion speed and motion transparency is labeled as “danger zone” in Figure 5n. These zones correspond to the entry and exit locations of narrow passages.

In sum, motion transparency appears in this scenario at the opening and exit of narrow passages due to the motion direction difference of 90° and for the side-view of a small-sized walker due the motion difference of the persons arm movements compared to the main body motion. A combination of transparency and motion speed as separate features largely resolves the ambiguity that the transparency signal alone has. In the example of a crowd passage through an exit shown in Figure 5d and 5e as well as 5j and 5k, it appears that motion transparency is necessary but not sufficient to indicate a “danger zone”. Only if the underlying motion of the transparent region appears in combination with slow motion speed, this indicates danger as is shown in Figure 5f and 5l. Locally and globally detected CON indicates the presence of a “danger zone”. Locally detected CON occurs in the proximity of the “danger zone” and is a precursor for dangerous behavior. Its interpretation is a slow-down of people flow. Although locally detected CON occurs in the spatial proximity of a “danger zone”, it is not fully aligned with the position of the “danger zone” in the image plane. Globally detected CON is not exactly localized due to the integration over large regions in the image. In our examples of Figure 5d and 5k the pattern motion of CON was detected for the center of the image, which roughly aligns with the detected “danger zones”, see Figure 5g and 5n. This larger CON motion pattern represents people’s motion towards a focus point, the focus of contraction. If only one such focus point is detected (unlike Figure 4d) people density increases. This increase in density causes further decrease in speed such that people can exit.

## Discussion

Various approaches exist in the literature that study the self-organization in behavior of people in crowds [1], [43], [44], that investigate the laws underlying such behavior [7], [45], and that analyze disastrous situations to extract characteristics and conditions that led to such situations [7], [44]. Besides physics-based modeling of behavior, several approaches automatically analyze videos for dangerous mass-behavior in motion crowds. Some of these approaches assume a resolution large enough for the detection of individuals, their representation by model shapes, and tracking over time (e.g., [6], [24]). Other approaches analyze the movement of pedestrians in videos of substantially lower spatial resolution [46], [47] or in parts substantially lower resolution using optic flow [48]. Our model detects and further processes visual motion as characteristic spatio-temporal “textures” [25], [26], [27]. This model is inspired by biological mechanisms of motion processing and is formulated in a cortical architecture that mimics core principles of an observer that routinely watches videos of people crowds. We suggest that the automatic analysis of motion patterns is improved by mechanisms that closely resemble human perceptual performance. Humans, like other primates, are capable to detect and represent motion transparency when multiple motions appear at a spatial image location. This is a property lacking in many computational vision approaches that detect and integrate motion (e.g., [35], [49], [50], [51], [52], [53], [54]). In addition, we have stressed that motion transparency occurs in non-dangerous as well as in dangerous situations. The distinguishing features were encoded in velocity gradients as well as in the spatial structure of the motion patterns. Such detection mechanisms have been specified in the proposed model and that were motivated by ample experimental evidence [15], [16], [19], [18], [19], [20]. In sum, we propose that a motion detector with a performance comparable to that of humans can routinely inspect videos like several human inspectors who watch the same videos. Several features and their combination are, in turn, indicative to mark potentially critical image locations as regions-of-interest for further more focused inspection by humans.

We emphasize here that we investigate only a subset of all potentially dangerous behaviors. In particular, we consider people crowds and focus on situations in which flow is jammed so that zero motion eventually occurs and that of merging people streams during an evacuation. In those situations the appearance of motion leads to transparent motion, a temporal reduction of motion speed, and characteristic spatial motion patterns. The analysis of such spatio-temporal configurations and combinations of it can be utilized to monitor public places, streets, or convention centers, and to signal the emergence of suspicious or potentially hazardous situations which could alert human inspectors to take a closer look at those indicated spots.

Our model has been described in full detail in [22], [42]. It incorporates different stages of processing related to different cells in areas closely resembling the functionality of visual cortex in primates. In a nutshell, the model consists of stages for initial motion detection and subsequent integration and segregation. Furthermore, in close relation to intermediate-level cortical processing large-scale cells, or filters, are employed that integrate the spatial patterns of input motion in different configurations. This model is not specifically adapted to a particular spatial resolution of the input and is, thus, able to process videos of different resolution including different details of peoples in crowds. One property different from many other models [30], [36], [49], [50], [51], [52], [53], [54] is the ability to process opaque motion and motion transparency which we suggest is important to fully characterize crowd motion at low spatial resolution. Our model explains various findings of perceiving motion transparency [22]. Processing of motion transparency is the key function that motivated us to apply the model framework to videos showing pedestrians or crowds in public spaces.

We suggest that motion transparency appears in crowd behavior. Lanes of opposite dot motions underlie a perceptual transition from segregated opaque motion organized in clearly distinguishable lanes into transparent layers of oppositely moving dots. This perceptual transition from opaque to transparent motion is a function of lane width [11]. Our model successfully explains the categorical perception of motion transparency generated by lanes of motion as well as other examples of motion transparency [22]. Therefore, we suggest that our model is capable to detect transparent motion for motion crowds in videos as would be perceived by human observers. To verify this suggestion we applied our model to scenarios of different crowd behaviors. We simulate motion crowds using the social force model [7] to acquire a reference. In addition, processing capabilities of our model for captured as well as rendered videos are demonstrated with publicly available videos. Captured video data shows non-dangerous crowd behavior in which people cross when they target different goal directions. If crowds move in opposite directions they form lanes of alternating motion directions of different widths depending on crowd density and available space. The variation of lane width leads to a perceptual transition of opaque motion to motion transparency, which depends on the crowd density and the spatial resolution of the sensor. Our model successfully handles this transition so that the motion representation is still capable of distinguishing non-dangerous from potentially dangerous situations, or a development thereof. Not all non-dangerous crowd behavior leads to motion transparency. When people target a single goal location and come from different directions so that a limited space has to occupy more and more people, then the crowd density increases. Depending on whether people can leave through an open funnel or cannot escape from this location several movement patterns occur. These patterns were detected by motion-sensitive cells that are selective to different characteristic patterns (such as contraction, clockwise or counterclockwise rotation, etc.) and by mechanisms that measure velocity gradients. Table 2 gives an overview to summarize motion crowds and their behavior.

**Table 2:** shows examples of coordinated and uncoordinated motion for dangerous and non-dangerous crowd behavior.

	Non-dangerous	Dangerous
**Coordinated motion** (e.g. highway, political rally, marathon, hallways, cheerleader dance)	Coherent single motion or coherent multiplemotions (Fig 3d–f and Fig 4)	Incoherent single motion or coherent multiple motions with slow speed (Fig 5)
**Uncoordinated motion** (e.g. crosswalks, waiting/exhibition hall, sidewalk)	Coherent multiple motions (Fig 3a–c, 3g–j,and Fig 2e)	No motion of people (Fig 2f) or incoherent single motion

In the following subsections we elaborate on crowded scenes and their coordinated or uncoordinated motion. We discuss cases of non-dangerous crowd behavior which exhibit motion transparency. We explain human perception of motion transparency, a motivation for our model development. Then, we discuss prior work in the context of our model and explain why the tracking approach is contrary to the concept of motion transparency. Finally, a fully automated model that includes learning and the integration of contextual cues is outlined.

### 4.1 Motion Transparency Occurs for Non-dangerous Crowd Behavior

Motion transparency appears for non-dangerous or common crowd behavior but not always. Several sequences of non-dangerous crowd behavior do not show motion transparency but coherent motion, e.g. when people exit in one direction. Here, we characterized a subset of crowd behavior that is described as incoherent due to the lack of an overall superimposed organization onto motions [24]. Examples of incoherent crowded scenes are the pedestrian motion at crosswalks, movements in a waiting/exhibition hall, or in general motion in an open area where individuals pursue approach of goals in different directions. In contrast, crowded scenes that appear coherent are moving cars on a highway, pilgrims at Mecca, runners in a marathon, or the cheerleaders following the choreography of a dance. All these examples share guidance or restriction of behavior in terms of a common objective or physical boundaries for the motion. For instance, individuals with a common objective could have the same driving, walking, or running direction that is constrained by external bounds, e.g. the highway, building, or runway.

### 4.2 Dangerous Crowd Behavior

In various contexts of crowded motion, non-dangerous versus dangerous behavior correlates with coherent versus incoherent motion, see also Table 2. Videos that are categorized to contain signs of dangerous behavior [55] show fights of peoples, or demonstrations that degenerate. In choreographed examples [56] dangerous behavior is characterized by faster uncoordinated and incoherent motion rather than zero motion. As pointed out above, in our analysis we describe a subset of all possible dangerous behaviors, that of a jammed flow which corresponds to zero motion and that of merging people streams during an evacuation that corresponds to the combined occurrence of motion transparency and slow motion speed. To monitor public places like sidewalks, waiting halls, etc. with low resolution this ‘no motion’-scenario of people seems a relevant case that is also produced by the dynamics of the social force model for pedestrian behavior [7].

We did not use captured videos of dangerous crowd behavior because this data is very rare and usually not publicly available [57]. For that reason we started our investigations by generating ground truth data under controlled conditions to study the different appearances of crowd behavior. Using the social force model [2], [40] we were able to define various spatial environments with different numbers of people (particles in the force nomenclature) as well as their movements. Such a parametrical study enabled us to generate non-dangerous as well as dangerous behaviors, such as clogging when a crowd is forced to leave through narrow passages. The important observation was that different behavior and transitions between them lead to appearance patterns in the crowd motion that are detected by the employed mechanisms in the model architecture, such as the detection of motion transparency, the detection of negative speed gradients, as well as motion patterns. In the social force model scenarios a birds-eye view position has been accomplished with a spatial resolution and distance where persons have a size of at least 10 pixels. This made the appearance more realistic than a simple point process for each particle. This spatial resolution allowed us to directly refer to the synthetic video data of crowds from [41] and real-life videos and to process them by using the same parameter setting in the model architecture.

In general, dangerous behavior depends on context [57], [58]. For instance, a running person in a mall could be a thief whereas the same person running outside on the sidewalk is more likely to be a jogger. In an airport it is even more ambiguous: There a running person can be either heading for the gate or trying to escape. These examples illustrate that the same behavior of running in different environments and settings can have different implications. This context-dependency challenges the design of automated systems. Certain local patterns of motion receive a different interpretation depending on the context that is defined by meta-information about the particular scene or event where the video is taken or the particular daytime. For this reason, we focused on the extraction and characterization of motions in videos and excluded the extraction of context information. However, our model is capable of including context-driven biases into the processing of motion. In a nutshell, those filter responses in the processing cascades that are compatible with a given motion direction or speed (feature) or which appear at a particular spatial position can be modulated and, thus, amplified (compare equation (6b) and the subsequent description). Such a modulation or attention bias leads to an enhancement of the corresponding filter activations such as their contrast would have been increased (compare [22], [59]). In sum, dangerous behavior depends on context. Our model allows for the integration of biases to model context information.

### 4.3 Our Model Relates to Human Perception of Motion Transparency

Multiple stimulus properties matter for the generation of a motion transparency percept: First, the relative spacing between stimulus points moving independently matters, while these points move back and forth temporally. For a vertical and horizontal spacing below 0.2° of visual angle, flicker motion is perceived, whereas dot spacing greater than 0.2° leads to motion transparency [60]. Second, the organization of stimulus parts at large matters. If stimulus parts are organized in lanes of alternating left/right motion direction with a width above 0.05° of visual angle, “striped motion” is perceived (i.e. opaque motion in lanes of opposite motion directions), if the lane width decreases below this critical width, motion transparency is perceived [11]. Third, the distance between motions in polar space matters. Below ≈20° angular difference, a single motion is perceived. This single motion is computed as the mean of the two presented motions. In the range of ≈30° to ≈120° the angular difference between the motions appears larger than its veridical value. For an angular difference above ≈120° their veridical value is perceived [38]. Fourth, the number of motions matters. Without directing attention to any motion direction, one randomly chosen motion direction out of three can be detected at a 75% rate. Because the motion direction is chosen by random, it is assumed that participants actually perceived all three motion directions. Directing attention to only one pre-cued motion direction allows for its correct detection or rejection among four others with a 75% rate [39]. Our model achieves similar results as humans for the latter three experiments [22]. In the context of the application to the processing of crowd behavior, these model computational results are useful as well. Since the majority of computational motion approaches cannot properly handle motion transparency, the disambiguation and separate representation of multiple motions provides an additional feature. Given the experimental data, an automatic mechanism could be parameterized so that it consistently analyzes scenes at a high level of attention priming. Human observers can keep such a level of focused attention for no more than 20 minutes. The model performance on the experimental data furthermore allows justifying which types of motion patterns the model architecture can deal with and thus serve as ground truth performance evaluation.

### 4.4 Relation to Prior Work

Our model presents a biologically inspired technique for the detection of dangerous crowd behavior which is an alternative to prior work as described in reviews [61], [62]. Abnormal motion behavior is characterized in [25] by using motion heat maps derived from regions with non-zero motion energy. These define regions-of-interest in which motion direction information is extracted. The classification of crowd motion patterns is based on the comparison of normalized histograms against reference distributions. Similarly the algorithm by Cao et al. [26] estimates optic flow from videos and analyzes the motion directions. Information theoretic measures for direction histograms in different image regions over time are used to indicate abnormalities in spatio-temporal patterns. Unlike these approaches, we do not rely on the distributions of motion information which accumulate motion estimates from a spatio-temporal region. Rather, we employ filtering and non-linear normalization computations in order to extract reliable motion features. We show the distributions of directional information but for local patches that appear opaque or transparent. Our normalization mechanisms can explain properties that a simple histogram based approach cannot replicate. Experimental evidence as discussed above suggests an interaction between motion components when perceiving motion transparency. Motion components of small angular difference are attracted and those of medium angular difference are repulsed. This can be explained by the employed center-surround interaction incorporated into our normalization mechanism. In addition, through the embedding of the normalization mechanism into feed forward and feedback signaling, the signal-to-noise ratio of temporally consistent motion components is enhanced as it essentially gathers “evidence” for motion components over multiple frames. Such interaction across frames is absent in the histogram based approach.

Yu and coworkers [27] proposed a scheme in which motion distributions (magnitude, direction) are segmented on the basis of treating motions as dynamic texture features. For different direction sets the motion energies are grouped over different spatial locations to extract dominant directional energy distributions. Such groupings also occur in our approach as a result of recurrent processing. Local competitive interaction among velocities allows for the grouping of noisy but similar motions into a single motion represented while the co-occurrence of two very different motions are segregated and represented as two motions. The motion patterns detected by selective cells in model MSTd may also be considered as textures of spatio-temporal appearances of motions in a given image region. These are, however, different from a static image that considers the local grouping directions as purely spatial arrangement. We argue that texture (and its spatial frequency content, or density) alone is insufficient to reliably detect and analyze crowd behavior since the temporal feature dimension is no longer available. Temporal changes in texture (or crowd) density are indicative for a potential development of hazardous situations (compare [2], [6], [8], [40]). In addition, by taking this temporal dimension, alias motion, into account allows for an analysis of the development of pictorial crowd patterns. The developments of motion contraction of the transition from transparent motion into an indistinguishable compressive flow pattern would be invisible in static textures. Other proposals [63] used a similar approach. This approach is based on the statistics of the local spatio-temporal motion gradients (mean and standard deviation) that are used by a Hidden Markov Model (HMM) to detect irregular motion patterns. Our method is based on the estimation of optic flow as well; however, we did not apply any (statistical) learning model atop of the extracted motion representation. Instead, we evaluated the computed velocity distribution based on its modality: A flat distribution relates to ‘no motion’, a unimodal distribution indicates the presence of a ‘single motion’, and a multimodal distribution corresponds to the simultaneous occurrence of ‘multiple motions’. The latter has been described as motion transparency.

Still other methods apply multi-tracking to follow many people of a crowd [24], [64]. Ali and Shah [64] included into their tracking algorithm forces defined by three fields. First, the static floor field has sink points at regions of attraction, e.g. exits. Second, the dynamic floor field encodes the behavior of all people within the neighborhood of oneself. Third, the boundary floor field is repellent at no-go spaces, e.g. walls. These three force fields are incorporated into a probabilistic framework for tracking. This approach is based on optic flow detection that does not account for motion transparency. Instead of tracking individuals in the crowd, we characterize regions of the image that contain a group of people with individuals moving in opposite direction as transparent. Furthermore, tracking of single pedestrians or detection of their outline requires some minimal size, often not provided in crowded motion data. For instance, available videos have a resolution of 480×360 pixels and pedestrians encompass approximately 10×10 pixels, but only blurred and combined with occlusions that lead to accretion and deletion between a pedestrian in the foreground and those in the background. Thus, a pedestrian of this size might be only partially visible at each time. Rather than trying to segment pedestrians and compute their entire shape from a low-resolution image we suggest that image motion in such videos is better characterized by motion transparency, where stimulus parts move independently but are in close spatial neighborhood. A multimodal distribution is generated when integrating these multiple coherent motions independently over a larger area of visual space. This distribution represents multiple motions and, thus, motion transparency.

Johansson et al. used image processing in combination with the social force model to automatically analyze video streams [6]. They assume a spatial resolution large enough to fit circular patterns, the head of people, to initially detected contrast edges. These circles are temporally tracked to estimate and represent the motion of people. This model is used to estimate crowd density, velocity, and flows and to identify critical indicators for the development of danger in crowd behavior. Johansson et al. [6] identified crowd pressure as a feature derived from combining the variance of speeds and density as signature for hazardous situations. Another sign of dangerous crowd behavior are stop-and-go-waves that suddenly appear within laminar motions. The appearance of slow-down is expressed by decelerations or a negative motion speed gradient in close proximity to regions of dangerous behavior that we identified by the combined presence of slow motion speed and motion transparency. Note that slow-down can also occur due to viewing perspective which has been suggested to correct for in [27]. In our model we did not aim for a transformation of the viewing perspective toward a top-down view assuming the image plane being fronto-parallel to the ground. Rather, we, suggest that our model provides motion features which may serve to highlight image regions of dangerous behavior. In a different context, this could be interpreted as a specialized attention mechanism that operates over the space and feature domain [65], [66] and which might provide an assistive tool for highlighting potentially dangerous crowd behavior to video inspectors.

### 4.5 Learning to Distinguish Dangerous from Non-dangerous Crowd Behavior

The motion representation that is extracted by our model enables learning to discriminate between security relevant and irrelevant behavior in a crowd of soccer fans. In the work of Endres et al. [57] the generation and labeling of data was performed in cooperation with experts. However, most other data available or produced did not include expert knowledge and the validity of its labeling is questionable. Lacking proper data we did not include learning in our modeling effort.

The motion representation extracted by our model can serve as basic feature for learning. Thereby, the spatial distribution of detected motions and their coherence among several frames are expected to be key discriminant features for detectors. However, such a proposed detector has yet to include context-dependent knowledge from other sources than visual motion.

### Conclusions

We suggested that motion transparency occurs in spatio-temporal patterns of motion crowds in non-dangerous and dangerous behavior. When motion transparency and low image speeds occur together, this indicates potentially dangerous behavior. We analyzed several videos showing non-dangerous and dangerous crowd behavior for which our model detected regions of motion transparency. The rationale to suggest a transfer of our biologically inspired model for visual motion processing came from its capability to explain various phenomena in human and animal motion perception. Our model is in agreement with several psychophysical [11], [39], [60] and physiological findings [67] which studied, for instance, stylized display configurations similar to lanes of opposite motion directions (compare simulation results in [22], [23]). Other configurations of motion transparency appear when random-dot patterns are overlaid moving in directions different enough to separate them.

In sum, we provide a visual front-end for processing video data as it relates to human perception of visual motion. We conclude that motion transparency in combination with low image speeds indicates dangerous behavior in motion crowds. Other motion features, such as locally defined speed gradients and globally defined motion patterns indicate where danger is emerging in motion crowds.

## Supporting Information

Figure S1 **Shows the simulation of velocity gradients for the scenarios of an open and partially closed walkway (a–d) and three real-life videos (e–j).** Details are explained in the supplement Text S1.
(TIF)

Figure S2 **Shows the simulated velocity gradients for the crosswalk of peoples at three levels of density. Details area explained in the supplement Text S1.**
(TIF)

Text S1 **Provides a description for the additional simulations of velocity gradients for the 1^st^, 2^nd^ and 3^rd^ scenario.** These scenarios are pedestrians walking on an open and partially closed walkway, real-life videos showing crowded motion, and people flows for a crosswalk at three levels of density.
(DOC)

## References

[1] Batty M (1997) Predicting where we walk. Nature, 388, 19–20. 10.1038/40266 9214493

[2] Helbing D, Johansson A (2010) Pedestrian, crowd and evacuation dynamics. Encyclopedia of Complexity and Systems Science, 16, 6476–6495.

[3] Cohen CJ, Morelli F, Scott KA (2008) A surveillance system for the recognition of intent within individuals and crowds. In: IEEE Conference on Technologies for Homeland Security, 559–565.

[4] Siebel NT, Maybank SJ (2004) The ADVISOR visual surveillance system. In: Proceedings of the ECCV 2004 workshop Applications of Computer Vision (ACV’04), 103–111. Prague, Czech Republic.

[5] Polus A, Schofer JL, Ushpiz A (1983) Pedestrian flow and level of service. Journal of Transportation Engineering 109(46), 46–56.

[6] Johansson A, Helbing D, Al-Abideen HZ, Al-Bosta S (2008) From crowd dynamics to crowd safety: A video-based analysis. Advances in Complex Systems, 11(4), 497–527.

[7] Helbing D, Farkas I, Vicsek T (2000) Simulating dynamical features of escape panic. Nature 407, 487–490. 10.1038/35035023 11028994

[8] Moussaid M, Helbing D, Theraulaz G (2011) How simple rules determine pedestrian behavior and crowd disasters. Proc. Nat'l Acad. of Science, 108(17), 6884–6888. 10.1073/pnas.1016507108 PMC3084058 21502518

[9] Qian N, Andersen RA, Adelson E (1994) Transparent motion perception as detection of unbalanced motion signals. iii. modeling. Journal of Neuroscience, 14, 7381–7392. 10.1523/JNEUROSCI.14-12-07381.1994 PMC6576878 7996183

[10] Zanker JA (2005) A computational analysis of separating motion signals in transparent random dot kinematograms. Spatial Vision, 18, 431–445. 10.1163/1568568054389615 16167775

[11] Burr D, McKee S, Morrone C (2006) Resolution for spatial segregation and spatial localization by motion signals. Vision Research, 46, 932–939. 10.1016/j.visres.2005.09.025 16289200

[12] Hubel DH, Wiesel TN (1970) Stereoscopic vision in macaque monkey. Cells sensitive to binocular depth in area 18 of the macaque monkey cortex. Nature, 226, 41–42. 10.1038/225041a0 4983026

[13] Ringach D (2002) Spatial structure and symmetry of simple-cell receptive fields in macaque primary visual cortex. Journal of Neurophysiology, 88, 455–463. 10.1152/jn.2002.88.1.455 12091567

[14] Born RT, Bradley DC (2005) Structure and function of visual area MT. Annual Reviews in Neuroscience, 28, 157–89. 10.1146/annurev.neuro.26.041002.131052 16022593

[15] Graziano MSA, Andersen RA, Snowden RJ (1994) Tuning of MST neurons to spiral motions. Journal of Neuroscience 14(1), 54–67. 10.1523/JNEUROSCI.14-01-00054.1994 PMC6576843 8283251

[16] Duffy CJ, Wurtz RH (1991) Sensitivity of MST neurons to optic flow stimuli. I: a continuum of response selectivity to large-field stimuli. Journal of Neurophysiology 65, 1329–1345. 10.1152/jn.1991.65.6.1329 1875243

[17] Duffy CJ, Wurtz RH (1995) Response of monkey MST neurons to optic flow stimuli with shifted centers of motion. Journal of Neuroscience 15(7), 5192–5208. 10.1523/JNEUROSCI.15-07-05192.1995 PMC6577859 7623145

[18] Xiao DK, Raiguel S, Marcar V, Koenderink J, Orban GA (1995) Spatial heterogeneity of inhibitory surrounds in the middle temporal visual area. Proceedings of the National Academy of Science USA, 92, 11303–11306. 10.1073/pnas.92.24.11303 PMC40620 7479984

[19] Xiao DK, Raiguel S, Marcar V, Orban GA (1997) The spatial distribution of the antagonistic surround of MT/V5 neurons. Cerebral Cortex 7(7), 662–77. 10.1093/cercor/7.7.662 9373021

[20] Duffy CJ, Wurtz RH (1997) Medial superior temporal area neurons respond to speed patterns in optic flow. Journal of Neuroscience 17(8), 2839–2851. 10.1523/JNEUROSCI.17-08-02839.1997 PMC6573103 9092605

[21] Raudies R, Neumann H (2010) A neural model of the temporal dynamics of figure-ground segregation in motion perception. Neural Networks 23, 160–176. 10.1016/j.neunet.2009.10.005 19931405

[22] Raudies F, Mingolla E, Neumann H (2011) A model of motion transparency processing with local center-surround interactions and feedback. Neural Computation, 23(11), 2868–2914. 10.1162/NECO_a_00193 21851277

[23] Raudies F, Neumann H (2012) Modeling binocular and motion transparency processing by local center-surround interactions. In Pomplun, M. and Suzuki, J. (eds.) Developing and Applying Biologically-Inspired Vision Systems: Interdisciplinary Concepts. IGI Global ISBN-10: 1466625392.

[24] Rodriguez M, Ali S, Kanade T (2009) Tracking in unstructured crowded scenes. In: Proceedings of the IEEE International Conference on Computer Vision, 1389–1396.

[25] Ihaddadene N, Djeraba C (2008) Real-time crowd motion analysis. In: Proc. IEEE Int'l. Conf. on Pattern Recognition, ICPR'08, Dec.8–11, 2008, Tampa, Florida, USA.

[26] Cao T, Wu X, Guo J, Yu S, Xu Y (2009) Abnormal crowd motion analysis. In: Proc. IEEE Int'l Conf. on Robotics and Biomimetics, ROBIO'09, Dec.19–23, 2009, Guilin, China.

[27] Yu H, He Z, Liu Y, Zhang L (2011) A dynamic texture-based method for multi-directional motions segmentation of crowd. In: Proc. IEEE 4th Int'l Congress on Image and Signal Processing, CSIP'11, Oct.15–17, 2011, Shanghai, China.

[28] Nyquist H (2002) Certain topics in telegraph transmission theory. In: Proceedings of the IEEE, “reprinted from Transactions of the A.I.E.E, 617–644, Feb. 1928”, 90, 280–305.

[29] Mechler F, Reich D, Victor J (2002) Detection and discrimination of relative spatial phase by V1 neurons. Journal of Neuroscience, 22, 6129–6157. 10.1523/JNEUROSCI.22-14-06129.2002 PMC6757932 12122074

[30] Hassenstein B, Reichardt W (1956) Systemtheoretische Analyse der Zeitreihenfolgen und Vorzeichenauswertung bei der Bewegungsperzeption des Rüsselkäfers, Chlorophanus. 2. Naturforschung Teil B, 11, 513–524.

[31] Stafstrom CE, Schwindt PC, Crill WE (2000) Receptive firing in layer V neurons from cat neocortex in vitro. Journal of Neurophysiology, 2, 254–277 (1984).S. Treue, K. Hol, and H.-J. Rauber. Seeing multiple directions of motion: Physiology and psychophysics. Nature Neuroscience, 3, 270–276. 10.1038/72985 10700260

[32] Angelucci A, Levitt J, Walton E, Hupe JM, Bullier J, et al.. (2002) Circuits for local and global signal integration in primary visual cortex. Journal of Neuroscience, 22, 8633–8646. 10.1523/JNEUROSCI.22-19-08633.2002 PMC6757772 12351737

[33] Albright T, Desimone R (1987) Local precision of visuotopic organization in middle temporal area (MT) of the macaque. Experimental Brain Research, 65, 582–592. 10.1007/BF00235981 3556486

[34] Albright T (1984) Direction and orientation selectivity of neurons in visual area MT of the macaque. Journal of Neurophysiology, 52, 1106–1130. 10.1152/jn.1984.52.6.1106 6520628

[35] Simoncelli EP, Heeger DJ (1998) A model of neuronal responses in visual area MT. Vision Research 38: 743–761. 9604103 10.1016/s0042-6989(97)00183-1

[36] Bayerl P, Neumann H (2004) Disambiguating visual motion through contextual feedback modulation. Neural Computation 16, 2041–2066. 10.1162/0899766041732404 15333206

[37] Durant S, Donoso-Barrera A, Tan S, Johnston A (2006) Moving from spatially segregated to transparent motion: a modeling approach. Biology Letters, 2, 101–105. 10.1098/rsbl.2005.0379 PMC1617172 17148338

[38] Braddick O, Wishart K, Curran W (2002) Directional performance in motion transparency. Vision Research, 42, 1237–1248. 10.1016/s0042-6989(02)00018-4 12044756

[39] Felisberti F, Zanker J (2005) Attention modulates perception of transparent motion. Vision Research, 45, 2687–2699. 10.1016/j.visres.2005.03.004 16022880

[40] Helbing D, Molnar P (1995) Social force model for pedestrian dynamics. Physics review E, 51(5), 4282–4286. 10.1103/physreve.51.4282 9963139

[41] Narain R, Golas A, Curtis S, Lin M (2009) Aggregate Dynamics for Dense Crowd Simulation. ACM Trans. Graph. 28, 5, Article 122.

[42] Raudies F, Ringbauer S, Neumann H Velocity gradients of optic flow locally encode ordinal depth at surface borders and globally they encode self-motion - An analytical and bio-inspired, computational model. In press. 10.1162/NECO_a_00479 23663150

[43] Xiong T, Zhang P, Wong SC, Shu CW, Zhang MP (2011) A macroscopic approach to the lane formation phenomenon in pedestrian counterflow. Chinese Physics Letters, 28(10), 108901-1/4.

[44] Moussaid M, Guillot EG, Moreau M, Fehrenbach J, Chabiron O, et al.. (2012) Traffic instabilities in self-organized crowds. PLoS Computational Biology, 8(3), e1002442, doi:10.1371/journal.pcbi.1002442. 10.1371/journal.pcbi.1002442 PMC3310728 22457615

[45] Henderson LF (1974) On the fluid mechanics of human crowd motion. Transportation Research, 8, 509–515.

[46] Saad A, Shah M (2007) A lagrangian particle dynamics approach for crowd flow segmentation and stability analysis. In Proceedings of Computer Vision and Pattern Recognition, CVPR’07, 1–6.

[47] Meheran R, Moore BE, Shah B (2010) A streakline representation of flow in crowded scenes. In Proceedings of the 11th European conference on computer vision conference on Computer vision, ECCV'10: Part III, 439–452.

[48] Krausz B, Bauckhage C (2012) Loveparade 2010: Automatic video analysis of a crowd disaster. Computer Vision and Image Understanding 116, 307–319.

[49] Heeger DJ (1988) Optical flow using spatiotemporal filters. International Journal of Computer Vision, 279–302.

[50] Adelson EH, Bergen JR (1985) Spatiotemporal energy models for the perception of motion. Journal of the Optical Society of America, Series A (2)2: 284–299. 10.1364/josaa.2.000284 3973762

[51] Lucas BD, Kanade T (1981) An iterative image registration technique with an application to stereo vision. In Proceedings of Image Understanding Workshop, 121–130.

[52] Horn BKP, Schunck BG (1981) Determining optical flow. Artificial Intelligence 17, 185–203.

[53] Papenberg N, Bruhn A, Brox T, Didas S, Weickert J (2006) Highly accurate optic flow computation with theoretically justified warping. International Journal of Computer Vision 67(2), 141–158.

[54] Zach C, Pock T, Bischof H (2007) A duality based approach for realtime TV-L1 optical flow. In F.A. Hamprecht, C. Schnörr, and B. Jähne (eds.): DAGM 2007, LNCS 4713, 214–223.

[55] Mehran R (2011) Videos of normal and abnormal crowd behavior. Available: http://www.cs.ucf.edu/%7Eramin/projects/abnormal_crowd/downloads/Normal_Abnormal_Crowd.zip, Accessed 2012 Aug.

[56] Mehran R, Oyama A, Shah M (2009) Abnormal crowd behavior detection using social force model. In: Proceedings of IEEE International Conference on Computer Vision and Pattern Recognition.

[57] Endres D, Neumann H, Kolesnik M, Giese MA (2011) Hooligan Detection: the Effects of Saliency and Expert Knowledge. In 4th International Conference on Imaging for Crime Detection and Prevention, ICDP-11, IET, 1–6.

[58] Jiang F, Wu Y, Katsaggelos AK (2009) Detecting contextual anomalies of crowd motion in surveillance video. In: Proc. IEEE Int'l. Conf. on Image Processing, ICIP'09.

[59] Bayerl P, Neumann H (2007) A neural model of feature attention in motion perception. BioSystems 89, 208–215. 10.1016/j.biosystems.2006.04.018 17280774

[60] Braddick O, Qian N (2001) The organization of global motion and transparency. In: J. Zanker & J. Zeil (Eds.) Motion vision: Computational, neural, and ecological constraints (p. 85–112). Heidelberg: Springer-Verlag.

[61] Hu W, Tan T, Wang L, Maybank S (2004) A survey on visual surveillance of object motion and behaviors. IEEE Transactions on Systems, Man, and Cybernetics - Part C: Applications and Reviews, 34(3), 334–352.

[62] Zhan B, Monekosso DN, Remagnino P, Velastin SA, Xu LQ (2008) Crowd analysis: A survey. Machine Vision and Applications 19: 345–357.

[63] Andrade EL, Blunsden S, Fisher RB (2006) Hidden Markov models for optical flow analysis in crowds. In: Proceedings of the IEEE International Conference on Pattern Recognition.

[64] Ali S, Shah M (2008) Floor fields for tracking in high density crowd scenes. In: Proc. of European Conference on Computer Vision, ECCV'08.

[65] Wolfe JM, Horowitz TS (2004) What attributes guide the deployment of visual attention and how do they do it? Nature Review Neuroscience, 5, 495–501. 10.1038/nrn1411 15152199

[66] Andersen SK, Hillyard SA, Müller MM (2008) Attention facilitates multiple stimulus features in parallel in human visual cortex. Current Biology, 18, 1006–1009. 10.1016/j.cub.2008.06.030 18595707

[67] Treue S, Hol K, Rauber HJ (2000) Seeing multiple directions of motion: Physiology and psychophysics. Nature Neuroscience, 3, 270–276. 10.1038/72985 10700260

