# Supplementary figures and images for: A Bio-Inspired, Motion-Based Analysis of Crowd Behavior Attributes Relevance to Motion Transparency, Velocity Gradients, and Motion Patterns
Source: PLoS One. 2012 Dec 31;7(12):e53456. doi: 10.1371/journal.pone.0053456 (PMC3534068; doi:10.1371/journal.pone.0053456)

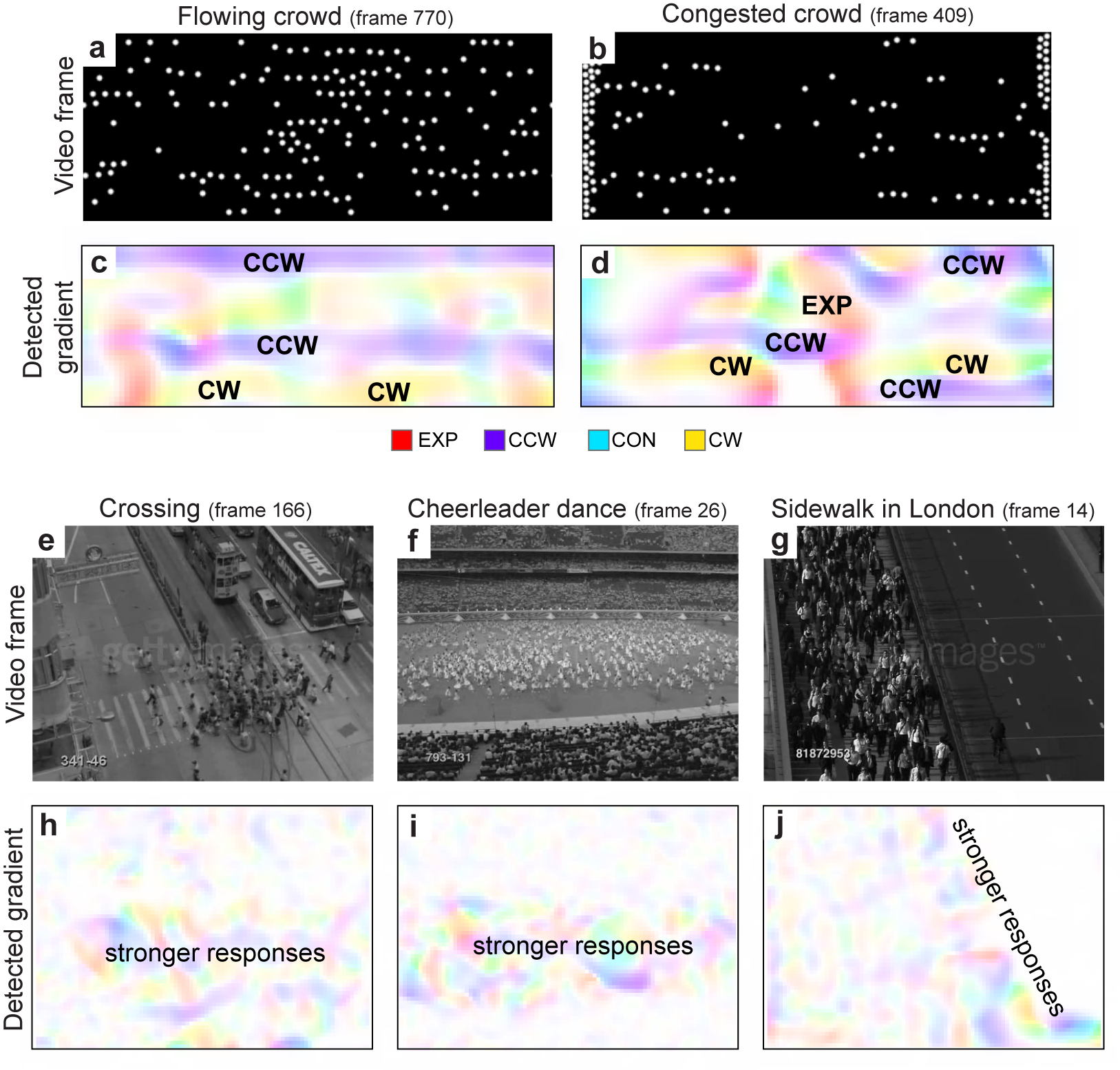

Supplement: Figure S1 — Shows the simulation of velocity gradients for the scenarios of an open and partially closed walkway (a–d) and three real-life videos (e–j). Details are explained in the supplement Text S1. (TIF) [file pone.0053456.s001.tif]

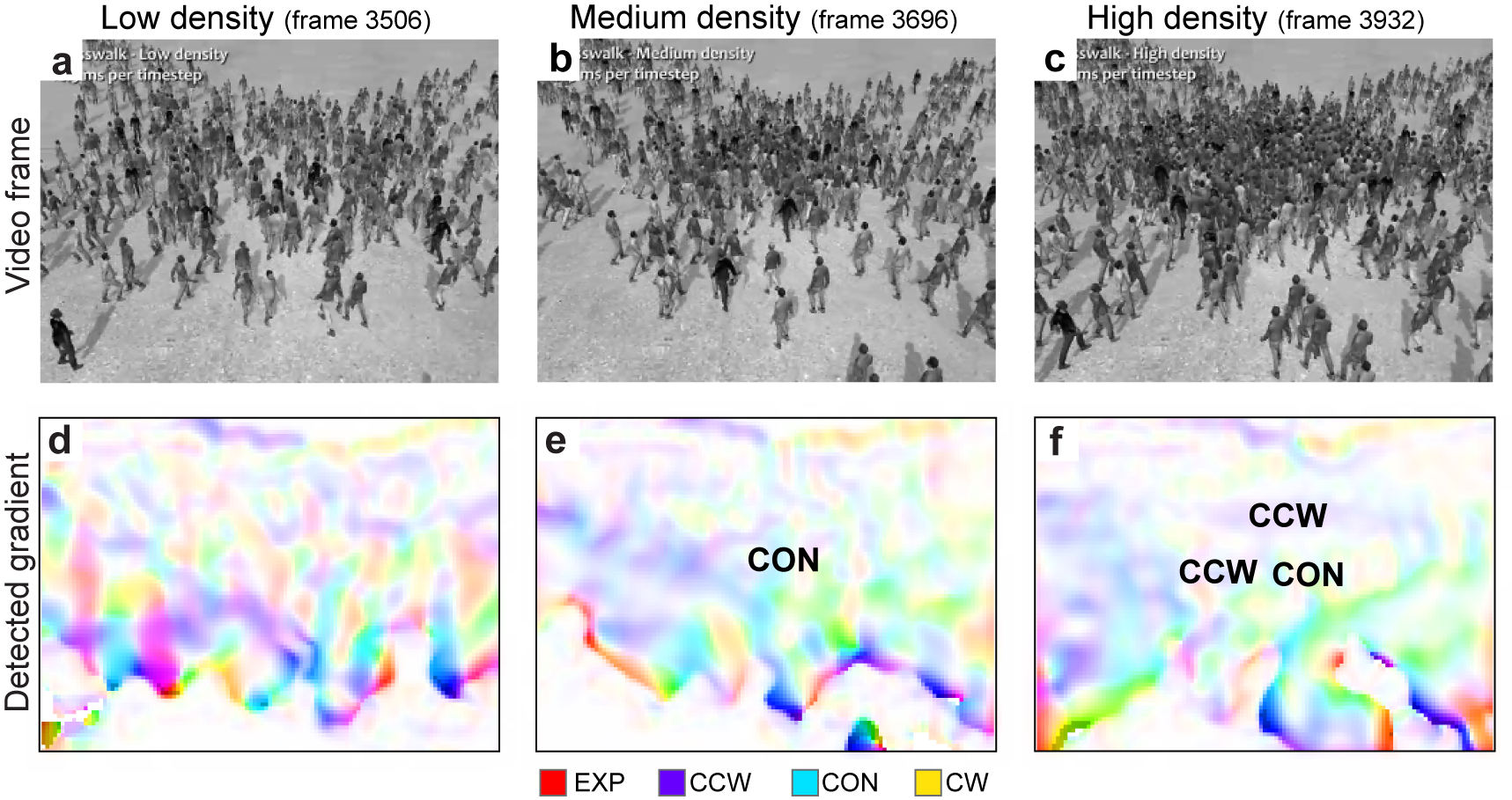

Supplement: Figure S2 — Shows the simulated velocity gradients for the crosswalk of peoples at three levels of density. Details area explained in the supplement Text S1. (TIF) [file pone.0053456.s002.tif]
