# Supplementary material for: A Bio-Inspired, Motion-Based Analysis of Crowd Behavior Attributes Relevance to Motion Transparency, Velocity Gradients, and Motion Patterns
Source: PLoS One. 2012 Dec 31;7(12):e53456. doi: 10.1371/journal.pone.0053456 (PMC3534068; doi:10.1371/journal.pone.0053456)
Supplement: Text S1 — Provides a description for the additional simulations of velocity gradients for the 1st, 2nd and 3rd scenario. These scenarios are pedestrians walking on an open and partially closed walkway, real-life videos showing crowded motion, and people flows for a crosswalk at three levels of density. (DOC) [file pone.0053456.s003.doc]

Raudies F, Neumann H A Bio-Inspired, Motion-Based Analysis of Crowd Behavior Attributes Relevance to Motion Transparency, Velocity Gradients, and Motion Patterns. *PLoS One* (in press)

**Supporting Information**

**Simulations for Velocity Gradient**

Here, we report the simulation results for velocity gradients for the remaining scenarios: the simulation of pedestrians walking on an open and partially closed walkway (Figure S1a – S1d), the processing of real-life videos showing crowded motion (Figure S1e – S1j), and the simulation of people flow for a crosswalk at three levels of density (Figure S2a – S2d).

For the walkway scenario the detected velocity gradients for the flowing crowd from Figure S1a shows an organization into four “stripes” visible in Figure S1c. These four stripes alternate between a CCW and CW local velocity gradient response. This resembles to some degree the velocity gradients at motion boundaries from Figure 5c (in the main paper). In Figure 5c CCW and CW responses appear in close proximity at the boundary between motion and “no motion”. Similarly, in Figure S1c, the velocity gradient has this CCW and CW response for boundaries between alternating motion directions. Thus, the velocity gradient contains still some information about the organization of motions into lanes while the displayed encoding of information from model area MT (Figure 2e) shows only the encoding into regions of “no motion”, “single motion”, or “multiple motions”. For the case of the partially closed walkway, where “multiple motions” largely disappear (Figure 2f) the velocity gradient encodes loosely organized motion lanes toward the ends of the walkway, again by responses of CCW and CW motion patterns at motion boundaries shown in Figure S1d. In addition, the central region encodes an EXP motion pattern since the few remaining dots in the center are moving to the left/right if they appear left or right from the center, respectively. Thus, the interpretation of velocity gradients is consistent with the one that we used for the explanation of simulation results in Figure 5c and 5j.

In the scenario of real-life videos the velocity gradient responses are largely absent, which Figure S1h-j shows. Note that we used the same scaling for the display of all velocity gradients. The only regularity between these three plots from Figure S1h-j is that velocity gradients are stronger for parts where the majority of visual motion appears in the image frame. In Figure S1h this is the lower half where the pedestrians walk on the crosswalk as Figure S1e shows. For the cheerleader video stronger velocity gradient responses appear over the full width of the image frame vertically organized around the center as displayed in Figure S1i. This region in the image corresponds to the area the cheerleaders perform their dance, see Figure S1f. In the last real-life video strong velocity gradient responses appear at the transition between “single motion” and “no motion” as indicated by the label “stronger responses” in Figure S1j. For the sidewalk in London as shown in Figure S1g, the strongest of the weak responses appears to the lower right end of the image frame due to the perspective, see Figure 5j.


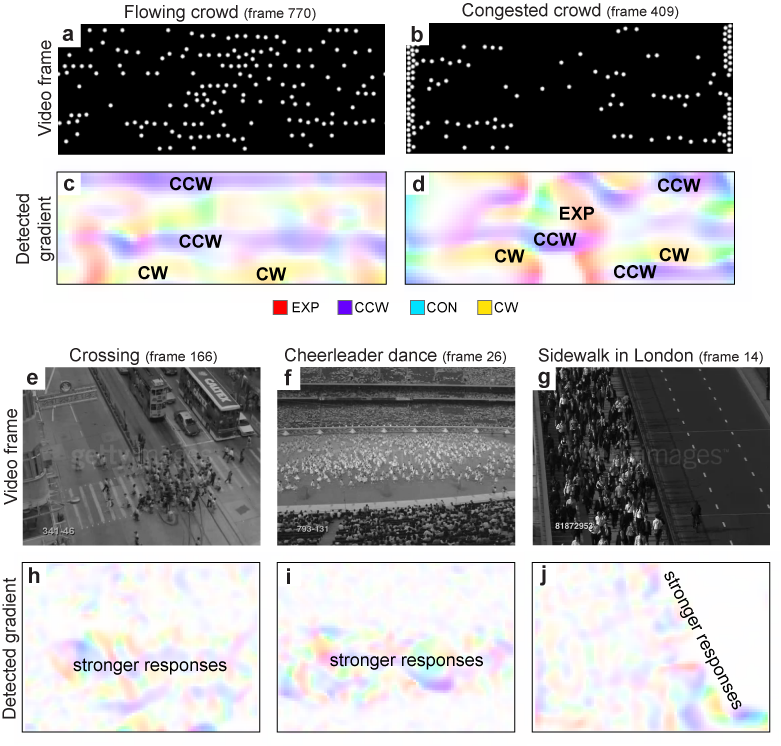


**Figure S1** shows the simulation of velocity gradients for the scenarios of an open and partially closed walkway (a-d) and three real-life videos (e-j). Details are explained in the text.

The third scenario studies the simulated people flow for a crosswalk at three density levels as Figure S2a-c show. For the low density case the velocity gradients are mainly unorganized as Figure S2d shows. At best, a loose organization into vertical stripes could be observed. This intermingled response of gradients largely corresponds to the detection of “multiple motions” as shown in Figure 4c. In general, this intermingled organization represents the intermingling of pedestrian flows. As before, velocity gradients appear strongest in their response at the lower part of the image frame where motions are faster due to the perspective. For an increase in density, regions with “multiple motions” are reduced, see Figure 4g. The central part in the image shows an CON velocity gradient as labeled in Figure S2e. For an even higher density this CON velocity gradient is accompanied by CCW responses as labeled in Figure S2f, which indicates the start of a CCW rotating stream of people in the intersection of the crosswalk. Note that the scaling for all color plots displaying velocity gradients is the same within and across figures.


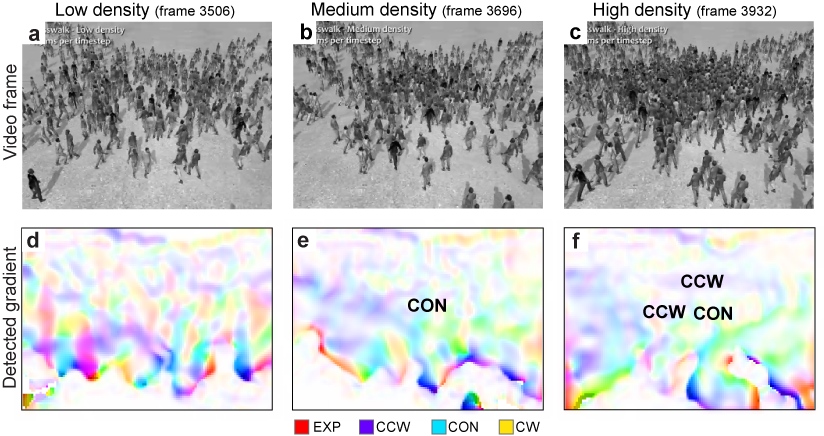


**Figure S2** shows the simulated velocity gradients for the crosswalk of peoples at three levels of density. Details area explained in the text.
